# Adherence and efficacy outcomes in young Australians with suicidal ideation using a self-management app and digital engagement strategy compared with a sham app: a three-arm randomised controlled trial

**DOI:** 10.1016/j.eclinm.2024.102963

**Published:** 2024-12-06

**Authors:** Michelle Torok, Lauren McGillivray, Daniel Z.Q. Gan, Jin Han, Sarah Hetrick, Quincy J.J. Wong

**Affiliations:** a University of New South Wales, Black Dog Institute, Sydney, Australia; b Black Dog Institute, Sydney, Australia; c Orygen Centre for Youth Mental Health, Parkville, Australia; d The University of Melbourne, Parkville, Australia; e New York University, Shanghai, China; f University of Auckland, Auckland, New Zealand; g School of Psychology, Western Sydney University, Sydney, Australia

**Keywords:** Suicide, Ideation, Self-harm, Digital, Adolescents, Young adults

## Abstract

**Background:**

Digital interventions are important treatment solutions for suicidal ideation, but premature disengagement is a significant threat to their effectiveness. We tested the adherence to, and efficacy of, two versions of an app-based intervention (app only, app + engagement strategy) for suicidal ideation, compared to a sham app.

**Methods:**

This was an online double-blind, three-arm parallel randomised controlled trial in Australia. Recruitment occurred between May 30 and August 8, 2023 and eligible participants were aged 17–24 years and had suicidal ideation in the prior 30 days. They were randomly assigned 1:1:1 to receive (i) LifeBuoy-an app which delivered third wave cognitive behavioural therapy (CBT) skills, (ii) the LifeBuoy app plus a digital engagement strategy, or (iii) a sham app to minimise expectancy bias. The primary efficacy outcome was change in suicidal ideation scores, measured by the Suicidal Ideation Attributes Scale (SIDAS), at 30–, 60– and 120– days post-baseline. The primary engagement outcome was the number of app modules completed at 60–days post-baseline. The final assessment occurred on December 6, 2023. All data was analysed using intention-to-treat. This trial was registered at anzctr.org.au, trial number: ACTRN12621001247864.

**Findings:**

692 participants were assigned (mean age: 19.9 [SD 2.5]; 70% female; intervention (combined): n–459, control: n–233). Significant reductions in ideation scores were observed in the combined intervention condition at 60– (d 0.48) and 120– (d 0.29) days after random assignment compared to the control condition. There were no differences in the number of modules completed between the intervention conditions (OR 1.10, 1.03, respectively) and no significant differences in their ideation scores at any time (ds −0.15 to 0.08). Serious adverse events (hospital presenting non-suicidal self-harm and/or suicide attempts) were reported by 6% of participants during the trial (control condition: 9%; combined intervention condition: 4%). No deaths were reported.

**Interpretation:**

A third wave CBT app helped to reduce ideation severity, however providing additional online resources to promote therapeutic engagement did not enhance these effects.

**Funding:**

This trial and MT was funded by the 10.13039/501100000925National Health & Medical Research Council, Matana Foundation for Young People, Alex Roth Foundation.

**Research in context**

**Evidence before this study**

We searched PubMed for studies from database inception to February 5th, 2024, for papers published in English including the following umbrella terms: “suicide”, “digital”, and “randomised controlled trial” (“RCT”). We identified five reviews examining small-scale, superiority RCTs of digital interventions for the self-management of suicidal thoughts and/or behaviours, which show small, time-limited effects for suicidal ideation but fail to demonstrate reductions in self-harm. Though digital interventions hold promise for reducing ideation severity, trials involving young people are rare. We identified no suicide prevention studies testing strategies to address the engagement challenges that characterise digital mental health interventions.

**Added value of this study**

This study investigated the efficacy, usage, and harms of an unsupervised, poly-therapeutic app based on third-wave CBT skills for the self-management of suicidal ideation. Exposure to the app was associated with significantly reduced suicidal ideation severity at 60- and 120-days, but not depression and anxiety symptoms or intentional self-harm. Providing regular psychoeducation materials and reminders adjunctively to the app did not increase app usage nor enhance its clinical benefits.

**Implications of all the available evidence**

As many adolescents and young adults with suicidal thoughts and behaviours experience barriers to accessing and engaging with traditional mental health services, self-guided therapeutic apps have potential to increase access to early intervention, potentially shortening the course of distress and preventing such thoughts being enacted. Therapeutic apps lend themselves to young people accessing earlier intervention, potentially shortening the course of distress and preventing such thoughts being enacted. This study demonstrates that if targeted to the problem, unsupervised digital interventions may support young people to develop skills that allow effective early intervention of suicidal ideation, addressing unmet treatment needs for this highly stigmatising condition. Further research is needed to understand how to design these innovations in ways that can achieve behavioural change and stronger effects.

## Introduction

Suicidal ideation is a clinically important indication for suicide attempts and suicide among young people^1^ with ∼60% of first attempts occurring within 12 months of ideation onset.^2^ Ideation is also linked to significant functional (executive) impairment,^3^ affecting individuals’ daily lives and future potential. Early intervention is needed to prevent these outcomes, however, adolescents and young adults with suicidal thoughts have some of the highest rates of unmet need for support. Much of this unmet need is driven by pervasive stigma and shame surrounding suicide and worries about what negative repercussions may result from disclosing ideation to a mental health clinician.^4^

Using common technology platforms to deliver targeted therapeutic support for suicidal ideation could be one solution for early intervention that overcomes the barriers that characterise face-to-face models of mental healthcare. Though trials in adult populations show that digital interventions can help to reduce the severity of suicidal ideation, their effectiveness and safety in young people is debated due to the low number of empirical studies currently available e.g.,^5^^,^^6^ Many young people express a strong preference for self-managing ideation and prefer to engage with psychological supports digitally.^7^ As such, self-directed apps that deliver proven behavioural therapeutic strategies to address risk processes linked to the onset and maintenance of ideation (e.g., emotion regulation, distress tolerance)^8^ may be a promising solution for young people unable or unwilling to engage with formal service provision. Our team previously developed a self-guided smartphone app (‘LifeBuoy’) for young adults with ideation in line with these assumptions based on poly-therapeutic third wave CBT approach (e.g., using techniques from dialectical behaviour therapy, acceptance and commitment therapy). A two-arm randomised controlled trial conducted during 2020 showed that this app led to small, short-term improvements in suicidal ideation compared to a placebo health information app.^8^ This contrasts with findings of another youth-specific app used a different variation of a third wave approach, more heavily focused on mindfulness, that failed to show superiority effects for ideation after six weeks of use.^9^ High quality trials to determine what therapeutic strategies may be active ingredients in the effective management of ideation are needed.

Though the acceptability of many digital mental health interventions is often high, trials typically show low rates of intervention completion, ranging between 0.5% and 28.6%.^10^ This acceptability–engagement paradox was observed in the prior LifeBuoy trial, and is a genuine and significant threat to the validity of effectiveness findings. While encouraging adherence is an important therapeutic and scientific goal, little is known about what strategies activate treatment adherence in respect to digital suicide prevention interventions and should be prioritised in their design and development.

As stated in our preregistered protocol,^11^ the co-primary aims of this three-arm randomised controlled trial were to investigate whether (i) the LifeBuoy intervention would be more effective than a sham app in reducing suicidal ideation intensity in adolescents and young adults, and (ii) whether a digital ‘engagement’ strategy delivered alongside LifeBuoy would lead to greater levels of app engagement (greater app module completion; greater number of app logins) compared to the LifeBuoy app alone. This study provides the first evidence in digital youth suicide prevention about specific approaches that may improve app engagement, and which allow us to infer effects of engagement on clinical outcomes. We also aimed to examine the effect of LifeBuoy on anxiety, depression, non-suicidal self-harm (NSSH), and suicide attempts over time, providing new insights into behavioural impacts and its potential harms. As LifeBuoy targets risk and protective processes shared with NSSH (i.e., emotional dysregulation, coping skills),^12^ we hypothesise it should prevent onset and reduce recurrence of NSSH. As NSSH is one the strongest predictors of transition to attempts among ideators, it may *indirectly* prevent suicide attempts.^13^

## Methods

### Ethics

All study procedures were approved by the University of New South Wales Human Research Ethics Committee (approval number: HC210400) and the study was conducted in accordance with the Declaration of Helsinki. The trial was pre-registered with the Australian and New Zealand Clinical Trials Registry, ACTRN12621001247864.

### Study design and participants

We conducted a double-blind, three-arm parallel randomised controlled trial, online, in Australia. Eligible participants were young people aged 17–24 years (inclusive) residing in Australia who had experienced suicidal thoughts in the past 30 days. They needed to own a smartphone (minimum version iOS-V.13 or Android-V.7) and be English-fluent. Participants were allowed to be engaged in psycho- or pharmaco-therapy, and information on concomitant treatment was collected at baseline. Main exclusion criteria were having received diagnosis of a psychotic or bipolar disorder in the 30 days preceding screening, as it would have been unlikely that such individuals would be on a stable treatment regime, potentially compromising their safety during, and adherence to, the trial.

### Randomisation and masking

Participants were randomly allocated (1:1:1) to one of the three conditions in block sizes of nine to receive LifeBuoy only, LifeBuoy plus access to a digital engagement strategy (LifeBuoy + Eng), or LifeBuoy-C, an attention matched “sham” app, providing an active comparison control. Random allocation was performed via a computer-generated algorithm integrated into the Black Dog Institute's bespoke automated trial management software (the ‘Research Engine’). App allocation decisions were communicated by receipt of a download link via email or SMS, and both the LifeBuoy and LifeBuoy-C apps were masked as ‘LifeBuoy’ in the Google Play and Apple app stores to maintain blinding. We also used a blind-to-hypothesis approach, in which prospective participants were informed they would be randomly assigned one of two apps under evaluation for the management of suicidal ideation, and that the digital therapies might include elements of education and behavioural self-management strategies and/or advice. Participants were not informed about randomisation probability, block size, or their assigned intervention condition. All study investigators, including the trial analyst, were masked to condition allocation during the trial.

### Procedures

All trial processes, including opt-in consent, were automated by the Black Dog Institute's bespoke digital trial platform and completed online. Participants were recruited online, via Black Dog Institute's social media channels (Instagram, Facebook, X). The advertisements contained a link to the trial portal, where interested individuals completed consent, screening, and trial registration. Individuals aged 17 years were required to pass a Gillick competence test^14^ in addition to the standard consent process to verify their understanding of the trial procedures. Participants were sent links to complete online surveys at baseline (T0), 30-days (T1), 60-days (T2), and followed up at 120-days (T3) post-baseline. They were compensated with $10 e-gift vouchers for completing surveys at T1–T3. Participants were sent their app link following baseline survey completion and had 7 days to download it before this link was disabled. Participants had access to their app until the T3 survey completion date.

At each survey, the research team were automatedly alerted via email if participants reported any known or serious adverse events (defined in Protocol S1). In parallel, participants were sent an email or SMS asking if they wished to be contacted by a clinical psychologist. If yes, this contact occurred by phone within 72 h of their response. All expected and serious adverse events were reported to the ethics committee and to our Data Safety Monitoring Board. Participants who reported a serious adverse event were withdrawn from the study if they said that it was attributable to the intervention or trial and contacted by the project psychologist to determine safety.

Both intervention conditions (LifeBuoy, LifeBuoy + Eng) were allocated LifeBuoy, a self-guided therapeutic smartphone app composed of seven structured modules (comprising 20 therapeutic activities) in which users learn and practice third wave CBT skills focused on emotion-regulation, distress tolerance, acceptance, values, and mindfulness, addressing key risk processes linked to ideation.^15^ The development of the original app is described elsewhere.^8^ Several modifications were made to the app following the first trial, based on participant feedback and in consultation with a youth lived experience advisory panel. The original seven modules were condensed into five modules and two new CBT-focused modules were added to better align the app to the stress-diathesis model. One new module focused on strengthening users’ internal locus of control and the other focused on managing stress. The original distraction activities, mood-monitoring function, and access to crisis helplines were retained, but a new self-care activity tracker and a sharable safety plan were added. We improved the journey logic so that users reporting a negative mood at check-in were directed to their safety plan, crisis contact numbers, and/or self-soothing activities. All modules could be completed within 10 min and were unlocked sequentially upon completion, and then remained unlocked. They received bi-weekly push notifications to complete tracking activities or access the toolbox and safety features, which they could disable. Participants were not given instructions on recommended dosage but were informed that they would have access to their app until their final assessment.

Though the LifeBuoy app did contain some integrated engagement features (i.e., push notifications [reminders to log in], self-monitoring, praise), the LifeBuoy + Eng condition were provided with access to additional strategies that have shown more promise in activating engagement in trials of digital health interventions: skills reinforcement and mastery, boosting beliefs about skill competencies, and affirming progress.^16^ The full development of the strategy is detailed elsewhere,^17^ but consisted of three digital components: (a) an Instagram account containing 42 posts, (b) an online blog consisting of 12 stories, and (c) weekly emails for up to 60 days post baseline reminding participants of these resources and where they could find different skills in the LifeBuoy app. Parts (a) and (b) of the strategy both focused on building knowledge of the benefits of practicing the same therapeutic skills included in LifeBuoy, example exercises, normalizing setbacks, and providing affirming messages to encourage ongoing practice and self-reflection. The two online platforms (Instagram, blogs) were selected for their ability to provide information in different levels of detail. To maintain participant blinding the Instagram channel was publicly accessible so that participants did not need to sign up to view it, and the public comment functionality was disabled. To minimise inadvertent exposure in other conditions, the Instagram channel was called “Remind Your Mind” and LifeBuoy was not explicitly mentioned in any posts. Following enrollment of the first participant in the LifeBuoy + Eng condition, one new piece of engagement material (e.g., Instagram post or blog) was posted every two to three days over the course of the trial.

The sham app (‘*LifeBuoy-C*’) provided an active comparison control as it looked and operated like LifeBuoy and was matched for task and time expectancy to control for digital placebo effects. The seven modules provide general information relating to positive mental health, including understanding stress, improving confidence, and the health benefits of having goals. In addition to these modules, the app included the same mood monitoring tracker, safety plan, and distraction activities as in LifeBuoy to allow for convincing participant-blinding.^8^ At the final survey, participants were given access to LifeBuoy for 30-days.

### Outcomes

All primary and secondary outcome measures were assessed at all timepoints. The primary efficacy endpoint was suicidal ideation in the past 30 days, measured using the Suicidal Ideation Attributes Scale (SIDAS).^18^ The SIDAS assesses the frequency, severity, controllability, and impact (interference with daily activities) of suicidal ideation. Total scores range from 0 to 50 (measured using a 11-point Likert scale), with higher scores indicating more severe ideation. Scores of ≥21 indicate high risk for suicidal behaviour. The scale demonstrated good internal consistency in this trial (α = 0.78).

App usage data were automatically recorded by the app and uploaded to central servers when the devices were connected to the Internet. Consistent with how similar trials measure engagement from a systems use perspective,^19^ the primary engagement outcomes were the number of modules completed (out of 7), which required completion of all activities within the module, and the number of app logins, which was defined as the total number of times modules and toolbox activities were attempted.

Secondary efficacy endpoints assessed suicide attempts, NSSH, and depression and anxiety symptoms. Participants were asked how many times they had: (i) attempted suicide and (ii) engaged in non-suicidal self-harm since the previous survey. If they indicated one or more times at the T3 survey, they were asked if the incident had occurred in the past 30-days. Binary ‘yes/no’ suicide attempt and non-suicidal self-harm variables were created from this data. Depression symptoms (past two weeks) were measured using the Patient Health Questionnaire-9 (PHQ-9),^20^ a reliable and valid nine-item measure of the severity of impact on mood and functioning (Cronbach's α = 0.83). The nine-item PHQ-9 has a total score range of 0–27, and ranges of ‘10–14’, ‘15–19’, and ‘20–27’ can be respectively interpreted as moderate, moderate-severe, and severe depression. The severity of generalised anxiety symptoms was measured using the seven-item Generalised Anxiety Disorder-7 (GAD-7),^21^ which also demonstrated good internal consistency (Cronbach's α = 0.84). Scores on the GAD-7 range from 0 to 21, with cut points of 5, 10, and 15 being interpreted as mild, moderate, and severe levels of anxiety.

### Statistics

A pre-specified analysis plan in our protocol (Protocol S1) governed analyses, unless identified as post hoc. We enrolled a total of 692 participants into this trial, which was sufficient to detect a between-condition effect size on the SIDAS of *d* = 0.45 or greater, and a between-condition effect size on engagement metrics of *d* = 0.38 or greater, with α = 0.01 and power = 0.95, and allowing for 25% attrition at our 30-day survey timepoint, based on previous studies.^8^^,^^22^^,^^23^ These original sample size calculations stated in our protocol were based on a repeated measures ANOVA model. Completing this, a summary-statistics-based power analysis for mixed models^24^ was conducted which indicated that our sample size would detect a between-condition effect for the SIDAS given *t* = −3.83 (*d* = 0.45)^8^ with α = 0.05, power = 0.80, and 25% attrition. A similar power analysis was not conducted for engagement metrics, as the engagement data could not be analysed as planned in our protocol, given consolidation of engagement data across timepoints. The negative binomial model analytical approach described in this section for engagement data should be regarded as post hoc. Nonetheless, the obtained sample size enabled detection of a 50% or larger increase in the count rate for LifeBuoy + Eng vs LifeBuoy using a negative binomial model (equivalent to between-condition effect size for engagement metrics of *d* = 0.38 or larger), with power = 0.80 and assuming a theta parameter as low as 0.70.^25^

Summary statistics were used to examine baseline differences between conditions on demographics (e.g., age, gender identify [female/male/non-binary, prefer not to say], sex assigned at birth [female/male]), clinical characteristics, and app engagement (intervention conditions only). Trial results were analysed for all randomised participants including those with missing follow-up data, representing the intention-to-treat population. Continuous primary and secondary outcomes across T0, T1, T2, and T3 were examined using linear mixed models for repeated measures analyses with maximum likelihood estimation. Three ‘Time’ variables (reflecting a piecewise approach for T0–T1, T1–T2, T2–T3), Condition (intervention conditions combined [LifeBuoy and LifeBuoy + Eng trial arms combined] vs LifeBuoy-C), and corresponding Time × Condition interactions were specified as fixed effects, and a random intercept for participants was included. An identity covariance matrix was employed, and degrees of freedom were estimated using Satterthwaite's method. Effect sizes (Cohen's *d*) were calculated based on relevant modelled mean differences and *SD*s at the relevant timepoints. Changes over time in binary secondary outcomes (yes/no) were analysed similarly but with generalised linear mixed models (GLMMs) using a binomial distribution and a logit link, together with maximum likelihood estimation. Effect sizes are presented as odds ratios (ORs). Post-hoc analyses were also conducted where the previous analyses were repeated but with the three trial arms as levels of the Condition variable (Condition: LifeBuoy vs LifeBuoy + Eng vs LifeBuoy-C) to allow each intervention condition to be compared with LifeBuoy-C. A similar analysis compared the two intervention conditions on the primary outcome, consistent with our protocol.

Post-hoc negative binomial regression analyses were used to compare LifeBuoy and LifeBuoy + Eng on the number of completed modules and the number of app logins. Effect sizes are presented as incidence rate ratios (IRRs). Post-hoc chi-square analyses were used to compare these conditions on other module completion metrics (completed all modules; did not complete any modules). In the case of significant differences in module completion or app logins between intervention conditions, post-hoc analyses examined these variables as potential mediators of changes in SIDAS scores.

Harms were described as the proportion of participants: (a) with SIDAS scores ≥21 at T1 through to T3 (known adverse event), (b) reporting a suicide attempt or episode of NSSH that required medical intervention (serious adverse event), and (c) if NSSH or a suicide attempt was directly caused by the intervention (serious adverse event). To model differences in variables (a) and (b) at each timepoint, GLMMs with the same approach as previously specified were conducted post-hoc.

Attrition analyses were used to identify any significant differences in the baseline characteristics between those who completed vs did not complete the T1, T2, and T3 surveys. Adjusted linear mixed models were run for SIDAS scores, controlling for any characteristics significantly differentiating completers from non-completers.

All continuous variables approximated normality (based on absolute skew and kurtosis) and ‘yes’ responses for binary outcomes ranged from 5.4% to 50%. For count outcomes, number of completed modules was left-skewed whereas number of app logins was right-skewed. For all linear mixed models examined, relevant residual plots and residual distributions indicated no notable concerns and suggested model assumptions were met. Alpha was set at ≤0.050 for statistical significance. SPSS Statistics 27.0 and the R package lme4^26^ were used for analysis.

### Role of the funding source

The funder and the sponsor had no role in the study design, data collection, data analysis, data interpretation, or writing of the report.

## Results

692 participants were recruited and randomly allocated between May 30 and August 8, 2023, and the final T3 assessment took place on December 6, 2023. Across the total sample, suicidal ideation (M 26.33, SD 10.00), depression (M 18.47, SD 5.32), and anxiety (M 13.34, SD 4.85) were all elevated at baseline, and 412 (59.5%) had attempted suicide at least once (lifetime) while 619 (89.5%) had engaged in NSSH (lifetime).

Sample characteristics were well balanced across the three study conditions (Table 1). Across the total sample, the mean participant age was 19.91 years (SD 2.52), 481 participants identified as female (69.5%), and 400 (57.8%) identified as lesbian, gay, bisexual, queer or other than heterosexual (LGBQ+). Almost all participants reported a history of mental ill-health (n = 662, 95.7%), and 436 (63.0%) were in receipt of pharmaco- and/or psychotherapy at trial registration.

**Table 1:** Participant characteristics at baseline.

	LifeBuoy (n = 230)	LifeBuoy + Eng (n = 229)	LifeBuoy-C (n = 233)
Age in years (M, SD)	19.93 (2.57)	20.09 (2.50)	19.73 (2.50)
Gender identity, n (%)			
Female	157 (68.3)	160 (69.9)	164 (70.4)
Male	32 (13.9)	35 (15.3)	32 (13.7)
Non-binary	37 (16.1)	30 (13.1)	30 (12.9)
Prefer not to answer	4 (1.7)	4 (1.7)	7 (3.0)
Sex assigned at birth, n (%)^a^			
Female	202 (87.8)	202 (88.2)	201 (86.3)
Male	27 (11.7)	26 (11.4)	32 (13.7)
Identifies as LGBQ+, n (%)	126 (54.8)	138 (60.3)	136 (58.4)
Education, n (%)			
High school qualification	133 (57.8)	136 (59.4)	155 (66.5)
Graduate certificate or diploma	68 (29.6)	59 (25.8)	39 (16.7)
University Degree	29 (12.6)	34 (14.8)	39 (16.7)
Living arrangements, n (%)			
With parents or other family	150 (65.2)	132 (57.6)	146 (62.7)
With partner, friends, or flatmates	60 (26.1)	74 (32.3)	61 (26.2)
Alone	14 (6.1)	12 (5.2)	20 (8.6)
Other	6 (2.6)	11 (4.8)	6 (2.6)
Not in intimate relationship, n (%)	150 (65.2)	127 (55.5)	150 (64.4)
Live in metropolitan area, n (%)	189 (82.2)	188 (82.1)	185 (79.4)
English language only speaker, n (%)	190 (82.6)	191 (83.4)	181 (77.7)
Employment status, n (%)			
Unemployed	31 (13.5)	27 (11.8)	26 (11.2)
Student (school, university)	117 (50.9)	114 (49.8)	124 (53.2)
Paid employment (casual, part-/full-time)	82 (35.7)	88 (38.4)	83 (35.6)
History of mental ill-health, n (%)	224 (97.4)	222 (96.9)	216 (92.7)
Ever seen a mental health professional, n (%)	194 (84.3)	200 (87.3)	191 (82.0)
Currently receiving mental health treatment (pharmaco- and/or psychotherapy), n (%)	147 (63.9)	144 (62.9)	145 (62.2)
BIS trait impulsivity scores (M, SD)	19.67 (4.59)	19.87 (4.49)	19.81 (4.86)
Age, onset of suicidal ideation (M, SD)	13.43 (2.97)	13.56 (3.22)	13.30 (3.09)
SIDAS suicidal ideation scores (M, SD)	25.86 (9.91)	26.45 (9.66)	26.67 (10.42)
PHQ-9 depression symptom scores (M, SD)	18.47 (5.36)	18.62 (5.18)	18.31 (5.43)
GAD-7 anxiety symptom scores (M, SD)	13.27 (4.85)	13.22 (4.78)	13.52 (4.93)
Suicide attempt in past 30 days, n (%)	19 (8.3)	17 (7.4)	16 (6.9)
NSSH in past 30 days, n (%)	110 (47.8)	106 (46.3)	109 (46.8)
Lifetime suicide attempt, n (%)	135 (58.7)	143 (62.4)	134 (57.5)
Lifetime NSSH, n (%)	206 (89.6)	205 (89.5)	208 (89.3)
Number of modules completed, Mdn (range)	4 (0–7)	5 (0–7)	–
Number of app logins, Mdn (range)	12 (1–84)	12 (1–92)	–

LGBQ+, Lesbian, Gay, Bisexual, Queer or other than heterosexual; BIS, Barratt Impulsivity Scale-Brief; SIDAS, Suicidal Ideation Attributes Scale; PHQ-9, Patient Health Questionnaire 9; GAD-7, Generalised Anxiety Disorder-7; NSSH, Non-Suicidal Self-Harm; M, Mean; SD, Standard Deviation; n, number; %, percentage.

a One participant in the LifeBuoy condition and one participant in the LifeBuoy + Eng condition did not provide a response for this variable.

**Fig. 1:**
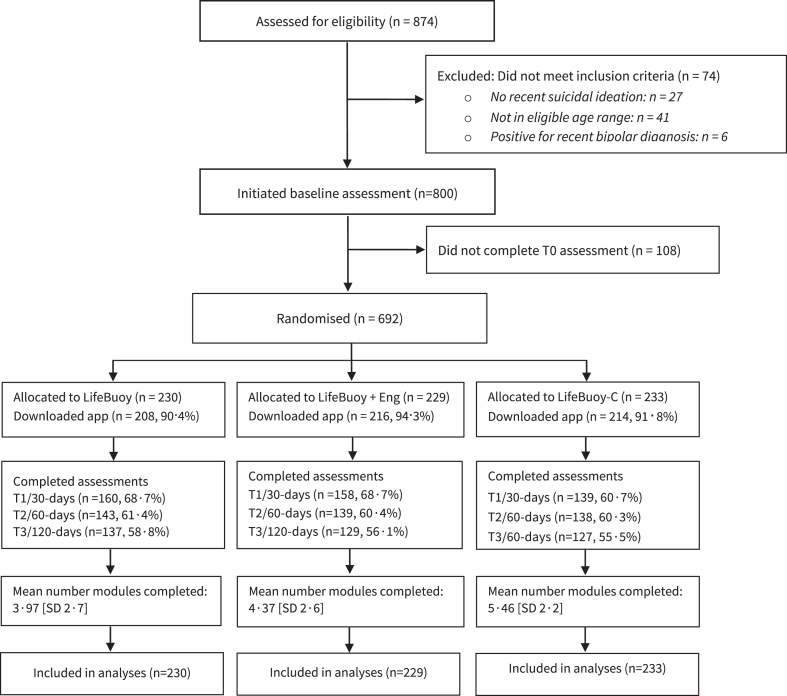
Trial profile.

Of the baseline sample, 457 (66.0%) completed outcome measurement surveys at T1, 420 (60.7%) at T2, and 393 (56.8%) at T3, and 638 (92.2%) participants downloaded their allocated app (see Fig. 1).

### Co-primary outcomes

**Fig. 2:**
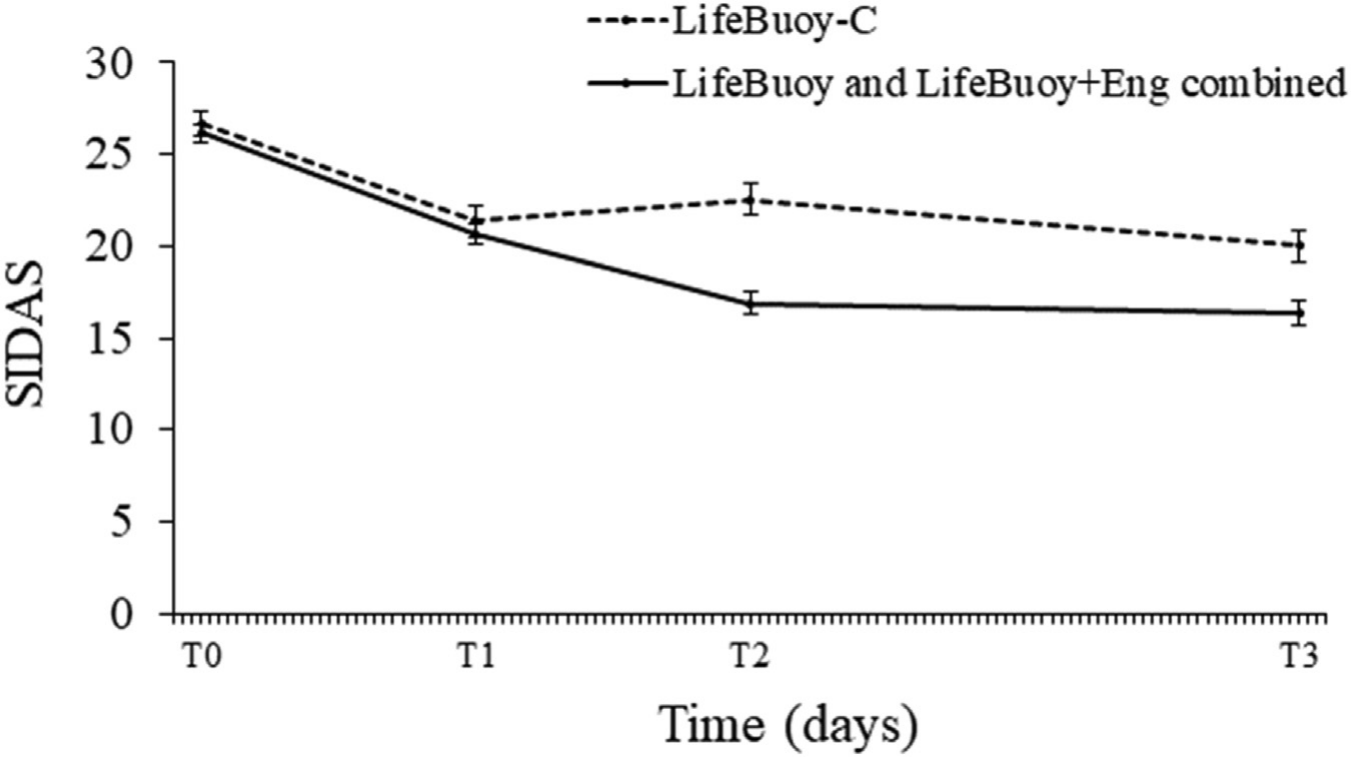
Modelled means on the Suicidal Ideation Attributes Scale (SIDAS) for LifeBuoy-C vs the combined intervention conditions (LifeBuoy, LifeBuoy + Eng) at baseline (T0) and post-baseline at 30-days (T1; p = 0.88), 60-days (T2; p < 0.001), and 120-days (T3; p < 0.001).

The model-based estimates and Time × Condition interaction effects for suicidal ideation are presented in Table 2. There was no significant difference between the intervention conditions combined and LifeBuoy-C in suicidal ideation severity from baseline to T1, but significantly greater reductions were observed for the intervention conditions combined from baseline to T2, and from baseline to T3 (see Table 2; Fig. 2). This resulted in significantly lower suicidal ideation scores for the intervention conditions combined relative to LifeBuoy-C at T2 (B = −5.69, 95% CI [−7.76, −3.62], t [1612.59] = −5.39, p < 0.001, d = 0.48) and T3 (B = −3.65, 95% CI [−5.76, −1.54], t [1658.01] = −3.40, p < 0.001, d = 0.29). Notably, the significant Time × Condition interactions in Table 2 were not moderated by receipt of pharmaco- and/or psychotherapy at trial registration (in additional models, both Time × Condition × current treatment interaction ps > 0.426). Additionally, a similar pattern of results was observed when each intervention condition was compared with LifeBuoy-C (see Text S1). There were no differential Time x Condition effects when comparing LifeBuoy + Eng with LifeBuoy on suicidal ideation severity at any time point (see Table S2), and no significant differences in their suicidal ideation at these time points either (see Text S2).

**Table 2:** Model-based estimates and standard error for primary and secondary outcomes at each time point for LifeBuoy and LifeBuoy + Eng interventions combined vs LifeBuoy-C.

	LifeBuoy and LifeBuoy + Eng intervention conditions combined				LifeBuoy-C control condition				30-day assessment (T1)			60-day assessment (T2)			120-day assessment (T3)		
									Time × Condition			Time × Condition			Time × Condition		
	T0	T1	T2	T3	T0	T1	T2	T3	statistic	*df*	p	statistic	*df*	p	statistic	*df*	p
	(n = 459)	(n = 297)	(n = 277)	(n = 256)	(n = 233)	(n = 160)	(n = 143)	(n = 137)									
*Primary outcome*																	
Ideation SIDAS, M (SE)	26.16 (0.52)	20.67 (0.60)	16.87 (0.62)	16.36 (0.63)	26.67 (0.73)	21.34 (0.83)	22.56 (0.86)	20.01 (0.87)	t = −0.15	1425.94	0.877	t = −5.13	1432.99	<0.001	t = −4.86	1472.73	<0.001
*Secondary outcomes*																	
Depression PHQ-9, M (SE)	18.54 (0.27)	15.96 (0.31)	15.34 (0.32)	14.39 (0.33)	18.31 (0.38)	16.03 (0.43)	16.21 (0.45)	15.76 (0.45)	t = −0.59	1426.65	0.557	t = −2.10	1433.48	0.036	t = −3.05	1472.46	0.002
Anxiety GAD-7, M (SE)	13.25 (0.24)	11.91 (0.28)	11.46 (0.29)	10.61 (0.29)	13.52 (0.34)	11.85 (0.38)	11.69 (0.39)	11.29 (0.40)	t = 0.75	1409.93	0.454	t = 0.11	1416.43	0.915	t = −0.47	1454.95	0.640
Suicide attempts in past 30 days, n (%)	35 (7.6)	25 (8.4)	20 (7.2)	13 (5.1)	13 (5.6)	15 (9.4)	12 (8.4)	7 (5.1)	z = −1.16	–	0.246	z = −1.38	–	0.169	z = −1.39	–	0.166
NSSH in past 30 days, n (%)	216 (47.1)	140 (47.1)	117 (42.2)	91 (35.5)	109 (46.8)	72 (45.0)	60 (42.0)	46 (33.6)	z = 0.33	–	0.739	z = 0.02	–	0.982	z = 0.19	–	0.849

Note. Time × Condition interactions reflect the period from baseline to the specified timepoint. SIDAS, Suicidal Ideation Attributes Scale; PHQ-9, Patient Health Questionnaire 9; GAD-7, Generalised Anxiety Disorder-7; NSSH, Non-Suicidal Self-Harm.

When comparing LifeBuoy + Eng to LifeBuoy, there were no between condition differences for the estimated number of app modules completed (4.37 vs 3.97, respectively; IRR = 1.10, z = 1.39, p = 0.163) or the estimated number of app logins (15.61 vs 15.20, respectively; IRR = 1.03, z = 0.33, p = 0.746). There were also no differences between the LifeBuoy + Eng and Lifebuoy conditions for app completion (completed all seven modules: n = 73, 31.9% vs n = 61, 26.5%, respectively; ꭓ^2^ (1) = 1.59, p = 0.207). 81/459 participants did not complete any modules across the two conditions, with a lower non-engagement rate in the LifeBuoy + Eng condition (LifeBuoy + Eng: n = 30, 13.1% vs LifeBuoy: n = 51, 22.2%; ꭓ^2^ (1) = 6.50, p = 0.011).

In the LifeBuoy + Eng condition, exposure to the engagement strategy was assessed at fortnightly intervals until 60-days post-baseline. The response rate was low at all measurement occasions, ranging between 13.1% and 15.3% of the 229 participants in this condition. 11.8% (n = 27) visited at least one component of the engagement strategy during the trial (Instagram: 11.4%, n = 26, 69.2% visited between 1 and 5 times; Blog: 4.4%, n = 10, 90.0% visited between 1 and 5 times).

### Secondary outcomes

**Fig. 3:**
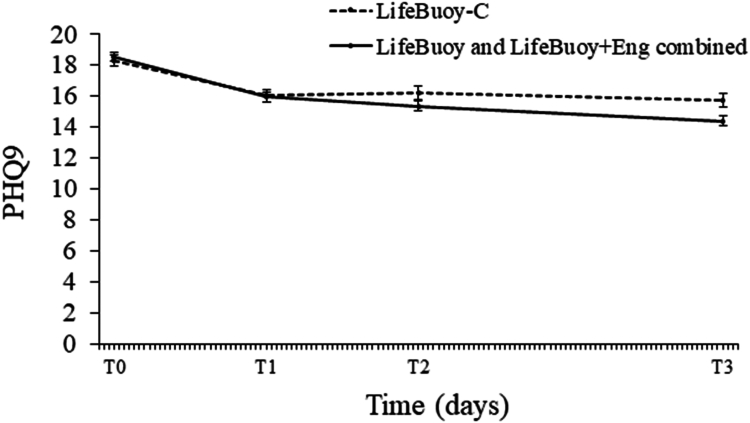
Modelled means of depression symptom scores (Patient Health Questionnaire-9; PHQ-9) for LifeBuoy-C vs the combined intervention conditions (LifeBuoy, LifeBuoy + Eng) at baseline (T0) and post-baseline at 30-days (T1; p < 0.56), 60-days (T2; p = 0.04), and 120-days (T3; p = 0.002).

There was no significant difference between the intervention conditions combined and LifeBuoy-C in the change in depression scores from baseline to T1, but there were significantly greater reductions in depression scores for the intervention conditions combined relative to LifeBuoy-C from baseline to T2, and from baseline to T3 (see Table 2; Fig. 3). Although there was no significant difference in depression scores for the two intervention conditions combined relative to LifeBuoy-C at T2 (B = −0.86, 95% CI [−1.94, 0.22], t [1602.68] = −1.57, p = 0.117, d = 0.14), there was a significant depression score difference favouring the intervention conditions combined at T3 (B = −1.37, 95% CI [−2.47, −0.28], t [1644.79] = −2.46, p = 0.014, d = 0.22). When the intervention conditions were each compared with LifeBuoy-C, LifeBuoy + Eng showed gains at T2 and T3, while LifeBuoy only showed gains at T3 (see Table S1a and b).

Scores for generalised anxiety significantly improved from baseline to T1, T2, and T3 in both conditions (Bs ranged from −2.64 to −1.34, all |ts| > 4.75, all ps <0.001, all ds ranged from 0.28 to 0.55), and no differential Time × Condition effects were evident (Table 2). A similar pattern of results was observed when each intervention was compared with LifeBuoy-C (see Table S1a and b).

There were no significant changes in the odds of participants who attempted suicide in the last 30 days from baseline to T1, T2 or T3 for either of the two conditions (ORs ranged from 0.38 to 3.09, all |zs| < 1.87, all ps > 0.062), and no differential Time × Condition effects evident (Table 2). Similar results were found when each intervention was compared with LifeBuoy-C (see Table S1a and b).

There were no significant changes in the odds of participants who engaged in NSSH in the last 30 days from baseline to T1 and T2 for either of the two conditions (ORs ranged from 0.74 to 1.01, all |zs| < 1.37, all ps > 0.170) and no differential Time × Condition effects evident (Table 2). At T3, both conditions had significantly reduced odds of participants who engaged in NSSH in the last 30 days relative to baseline (ORs ranged from 0.44 to 0.49, both |zs| > 2.66, both ps <0.008) with no differential Time × Condition effect (Table 2). Similar results were found when each intervention was compared with LifeBuoy-C (see Table S1 and b).

### Harms

Harms are reported as per the Consolidated Standards of Reporting Trials (CONSORT) harms statement^27^ and according to our trial protocol. Participants in the combined intervention condition had a significantly lower odds of being categorised as at ‘high risk’ for suicide attempt on the SIDAS (i.e., scores of ≥21) compared to the control condition at T2 only. A number needed-to-treat (NNT) analysis showed that n = 6 participants would need to be treated to prevent 1 additional person from scoring ≥21 on the SIDAS (Table 3).

**Table 3:** Model-based estimates of known and serious adverse events across time.

Timepoint	LifeBuoy and LifeBuoy + Eng interventions combined (n, %)	LifeBuoy-C (n, %)	OR^a^ [95% CI]	z	p	NNT^b^ [95% CI]	Absolute risk reduction %^b^ [95% CI]
**SIDAS scores ≥21 (high risk), n (%)**							
T0^c^	337 (73.4)	175 (75.1)	0.89 [0.52, 1.52]	−0.43	0.667	–	–
T1	135 (45.5)	74 (46.3)	0.96 [0.53, 1.76]	−0.13	0.901	–	–
T2	99 (35.7)	77 (53.8)	0.35 [0.19, 0.67]	−3.19	0.001	6 [4, 13]	18.11% [8.18%, 28.04%]
T3	74 (28.9)	53 (38.7)	0.55 [0.28, 1.07]	−1.77	0.077	–	–
**Suicide attempt or NSSH requiring medical intervention since last survey, n (%)**							
T0^c^	142 (30.9)	68 (29.2)	1.12 [0.63, 2.01]	0.39	0.696	–	–
T1	6 (2.0)	8 (5.0)	0.28 [0.09, 0.89]	−2.16	0.031	34 [15, ∞]	2.98% [−0.76%, 6.72%]
T2	6 (2.2)	8 (5.6)	0.27 [0.08, 0.92]	−2.10	0.036	30 [14, ∞]	3.43% [−0.71%, 7.57%]
T3	8 (3.1)	8 (5.8)	0.40 [0.13, 1.25]	−1.58	0.114	–	–

Estimated values (n, %) are relative to the following sample sizes: (a) LifeBuoy and LifeBuoy + Eng interventions combined: T0 n = 459, T1 n = 297, T2 n = 277, T3 n = 256; (b) LifeBuoy-C: T0 n = 233, T1 n = 160, T2 n = 143, T3 n = 137. T0 = Baseline; T1 = 30-day assessment; T2 = 60-day assessment; T3 = 120-day assessment; NNT, number needed to treat; SIDAS, Suicidal Ideation Attributes Scale.

a Reflects odds of being flagged as high risk on SIDAS or requiring medical intervention following suicide attempt or NSSH event for LifeBuoy and LifeBuoy + Eng interventions combined relative to LifeBuoy-C.

b NNT and absolute risk reduction provided only for timepoints where there was a significant difference between conditions.

c In 30 days prior to baseline.

Across the trial period, a total of n = 82 serious adverse were events reported by 42 unique participants (6.1% of total sample), defined as presenting to hospital for a suicide attempt or NSSH (suicide attempts: n = 45, 54.9%; NSSH: n = 37, 45.1%). Serious adverse events were most common in the LifeBuoy-C condition (n = 50, 61.0%) (see Table S3). The odds of a suicide attempt or NSSH requiring medical intervention was significantly less for the intervention conditions combined relative to the control condition at T1 and T2 (Table 3). No participants reported that their self-harm episode/s were directly caused by the apps or trial participation.

N = 11 incident (new) cases of suicide attempt were reported during the trial (LifeBuoy: n = 2 [both at T1]; LifeBuoy + Eng condition: n = 5 [T1: 1, T2: 3, T3: 1]; LifeBuoy-C: n = 4 [T1: 1, T2: 1, T3: 2]). N = 17 incident cases of NSSH were also reported (LifeBuoy: n = 3 [T1: 2, T3: 1]; LifeBuoy + Eng: n = 4 [T1: 3, T3: 1]; LifeBuoy-C: n = 10 [T1: 4, T2: 4, T3: 2]).

Over the course of the trial, there were no contact requests from participants to speak with the project clinical psychologist. No deaths were reported to the research team.

### Survey attrition sensitivity analysis

Univariate analyses of baseline characteristics were conducted to identify predictors of survey attrition (see Table S4a). The primary outcome analysis was repeated with adjustment for variables differentially associated with attrition at T1, T2, and T3, and results were unchanged (see Table S4b).

## Discussion

Results of the LifeBuoy randomised controlled trial show that a digital intervention based on a poly-therapeutic third wave CBT approach effectively and progressively reduced symptom severity of suicidal ideation over the course of the trial, and the change was statistically greater than that observed in relation to a sham app. The results on our co-primary ‘engagement’ outcome were null, highlighting that receiving information which reinforced and reminded participants of the nature and benefits of app's therapeutic content was not effective in increasing engagement with, nor the efficacy of, the LifeBuoy app. The odds of requiring medical care for self-harm was significantly lower in the intervention conditions, and no serious adverse events directly related to the intervention. Our results support the low risk nature of our interventional approach and show the strong benefit–risk profile of LifeBuoy.

The LifeBuoy app is one of few digital interventions shown to reduce ideation in young people. The superiority effect did not emerge until 60 days post-baseline (*d* = 0.48) and was sustained at a lesser magnitude until the 120 day assessment (*d* = 0.29). Both the temporality and magnitude of the change in ideation symptoms are consistent with the patterns observed at 42 days post-baseline in our previous pilot trial,^8^ not only corroborating the efficacy of the LifeBuoy app but also indicating that skills for the effective self-management of ideation may take a minimum of six weeks to develop.

With its therapeutic focus on confronting difficulties in emotional regulation and poor distress tolerance via explicit teaching of adaptive coping skills, LifeBuoy is differentiated from other trials of digital interventions for suicide prevention which typically provide psychoeducation and/or cognitive restructuring techniques targeting negative affect.^6^ With many of these trials having shown null effects,^6^ our primary outcome results suggest that developing emotion regulation self-efficacy may be an essential ingredient in the effective management of ideation for young people. Though our approach appears to be promising for unsupervised delivery models, even better outcomes may be achieved by integrating artificial intelligence personalisation algorithms to recommend—and engage users in–an optimal set of therapeutic strategies based on their unique risk profile and individual preferences.

A key challenge this study sought to overcome was how to improve engagement with digital health interventions, to reduce the threat that attrition poses to efficacy and validity. Though we co-designed our engagement with young people who represent the intended beneficiaries of LifeBuoy^17^ in an attempt to meet their needs, only one in eight participants engaged with the strategy, mostly infrequently. The low engagement with, and ineffectiveness of, the strategy is likely to be at least partially related to the fact that it did not explicitly link back to the LifeBuoy app. This approach was necessary to manage contamination risk but may have unintentionally diminished the relevancy of the strategy to LifeBuoy users. Having an engagement strategy that sat completely externally to the core intervention (LifeBuoy) may have also required too much psychological effort to access and interact with. Integrating engagement features (e.g., self-monitoring, rewards, customisation, gamification) into the app itself may be a more promising, less cognitively taxing, approach. A recent review of trials of mental health apps shows that the more persuasive design features that are included directly within digital health interventions, the larger the clinical effects.^28^ Rapid developments in artificial intelligence and design thinking are creating ever evolving opportunities to optimise app design to promote engagement in ways that reduce the cognitive load on end users (e.g., chat bots, personalisation features).

Contrary to our *a priori* hypotheses, there were no differential effects between conditions for secondary outcomes related to anxiety, self-harm, or suicide attempt, at any time point. A small difference was detected in depression symptom scores favouring the intervention at 120 days post-baseline, which was not observed in our prior trial.^8^ We argue that many of these null findings are the likely consequence of a “logic mismatch” between the core activities of LifeBuoy and our measurement design. Put simply, we assessed outcomes that the intervention was not designed to activate change in. LifeBuoy was purpose–designed to target risk processes theoretically- and empirically-linked to the pathology of suicidal ideation (e.g., emotion dysregulation, low distress tolerance)^12^ but which do not explain or attenuate the transition to suicide attempt (e.g., pain sensitivity, death mental imagery)^15^ nor the onset or maintenance of anxiety (e.g., high rumination, threat biases).^29^ Though emotion dysregulation is a common and important feature to both depression and NSSH, there was no convincing evidence that LifeBuoy led to clinically meaningful changes in these outcomes. Our null findings for NSSH are highly consistent with the results of numerous interventional studies, many of which have delivered much more potent courses of treatment (i.e., longer duration, higher frequency sessions).^30^ Not only is the brevity of LifeBuoy incongruous with the goal of achieving complex behavioural change, our sample size was underpowered by approximately 10-fold per condition to detect change in repeat self-harm.^31^ Our primary and secondary findings point to the need to ensure that digital intervention activities are *a priori* conceptually well-aligned to condition-specific risk processes, to at least have the opportunity to be effective, and the evaluation approach should adhere to this same pre-hypothesised logic.

A major strength of this study is the inclusion of a digital placebo to control for the expectation of success on efficacy, and the simultaneous use of trial automation software that enabled double-blinding conditions. With our three-arm design, this trial represents one of the first rigorous scientific efforts to disentangle the effect of a targeted engagement strategy from a therapeutic suicide prevention app on adherence and clinical outcomes.

The results of this trial should also be considered in the context of its limitations. Our rates of survey attrition at T1–T3 were high (i.e., 34%–43%), yet these rates are typical of most digital mental health trials (i.e., adjusted pooled rate of attrition is estimated to be 47%).^32^ Though sensitivity analyses of the primary endpoint showed that our results were unchanged by any factors associated with attrition, they are still likely overestimated. Females and young people identifying as LGBQ+ were overrepresented (70% and 58% respectively) as they tend to be in mental health trials, limiting how representative our findings may be of the broader population of young people who develop suicidal ideation. Nonetheless, these are groups who experience some of the highest rates of self-harm and ideation during adolescence and who should be prioritised in early intervention efforts. As the trial was internet-based, recruitment was self-selective, and we were unable to verify if participants genuinely met study inclusion criteria. Although registration data was periodically checked by the research officer, there are increased reports of fraudulent participants in online trials.^33^ Our results for suicide attempt or NSSH requiring medical intervention may be subject to sparse data bias due to their low prevalence (ranging from n = 8–13 incidents [1.7 & −5.6%] at follow-up timepoints) and should interpreted with caution.^34^

The findings from this trial highlight the potential of a low-intensity, third wave CBT app, like LifeBuoy, to increase young people's access to early intervention for suicidal ideation, safely. There was no convincing evidence of benefits beyond suicidal ideation, consistent with its conceptualisation, and offering additional engagement strategies did not improve app uptake nor its benefits. To better understand the active ingredients of LifeBuoy, a separate dismantling paper examining the relationship between different app modules and ideation symptom scores will be published. This future work, along with the mixed results reported in this trial may stimulate new research in several important areas for suicide prevention. These include improving the integration of ideation-to-action theory in app development, identifying what therapeutic approaches work best to reduce ideation and self-harm, and testing the use of novel synthetic trial designs to generate very large, well powered samples to overcome current innovation, conceptualisation, and methodological challenges that plague digital suicide prevention.

## Contributors

QW and MT had full access to all data in the study and take responsibility for verification of the data, and the accuracy of the data analysis. MT and QW also developed the initial outline and draft of the paper, which all authors contributed to. LM and DZQG led the literature search and review. QW led the statistical analysis of this data reported in this study. All authors contributed to the design, analysis, and conduct of the study and interpretation of the data. MT, LM, DZQG, and JH had key roles in the design and development of LifeBuoy and the engagement strategy. All authors read and approved the final version of the manuscript and take responsibility for the decision to submit for publication.

## Data sharing statement

Individual participant data that underlie the results reported in this Article, after deidentification (text, tables, figures, and appendices), will be shared by the corresponding author (m.torok@unsw.edu.au) on reasonable request for academic and research purposes and subject to data sharing agreements. The clinical trial protocol, including statistical analysis plan, is available in the supplementary materials.

## Declaration of interests

We declare no competing interests.

## Appendix A Supplementary data

Supplemental Data

Supplemental Data S1 Protocol

CONSORT checklist
