# Supplementary material for: Adherence and efficacy outcomes in young Australians with suicidal ideation using a self-management app and digital engagement strategy compared with a sham app: a three-arm randomised controlled trial
Source: eClinicalMedicine. 2024 Dec 6;79:102963. doi: 10.1016/j.eclinm.2024.102963 (PMC11665678; doi:10.1016/j.eclinm.2024.102963)
Supplement: Supplemental Data [file mmc1.docx]

Contents

[Supplemental Appendix 1. 2](#_Toc181695257)

[Analyses of primary and secondary outcomes comparing each intervention condition with LifeBuoy-C 2](#_Toc181695258)

[Supplemental Table S1a. Model-based estimates and standard error for primary and secondary outcomes at each time point for LifeBuoy vs LifeBuoy-C 4](#_Toc181695259)

[Supplemental Table S1b. Model-based estimates and standard error for primary and secondary outcomes at each time point for LifeBuoy+Eng vs LifeBuoy-C 5](#_Toc181695260)

[Supplemental Appendix 2 6](#_Toc181695261)

[Analyses of primary outcome comparing the two intervention conditions: LifeBuoy to LifeBuoy+Eng 6](#_Toc181695262)

[Supplemental Table S2. Model-based estimates and standard error for primary outcome at each time point for LifeBuoy+Eng vs LifeBuoy 7](#_Toc181695263)

[Supplemental Table S3. Serious adverse events x condition 8](#_Toc181695264)

[Supplemental Appendix 3 9](#_Toc181695265)

[Sensitivity analysis – primary analysis adjusting for variables associated with attrition 9](#_Toc181695266)

[Supplemental Table S4a. Variables associated with survey attrition (survey attrition sensitivity analysis) 10](#_Toc181695267)

[Supplemental Table S4b. Sensitivity analysis – primary analysis adjusting for variables associated with attrition 12](#_Toc181695268)

# Supplemental Appendix 1.

# Analyses of primary and secondary outcomes comparing each intervention condition with LifeBuoy-C

*SIDAS*

There was no significant difference between LifeBuoy and LifeBuoy-C in the change in suicidal ideation severity from baseline to T1, but there were significantly greater reductions in the severity of suicide ideation for LifeBuoy relative to LifeBuoy-C from baseline to T2, and from baseline to T3 (see Supplemental Table S1a), resulting in significantly lower suicidal ideation scores for LifeBuoy relative to LifeBuoy-C at T2 (B = -4∙84, 95% CI [-7∙24, -2∙45], t[1612∙55] = -3∙97, p < ∙001, d = 0∙39) and T3 (B = -4∙13, 95% CI [-6∙58, -1∙69], t[1664∙23] = -3∙32, p < ∙001, d = 0∙33).

A similar pattern of results was found when comparing LifeBuoy+Eng with LifeBuoy-C. There was no significant difference between LifeBuoy+Eng and LifeBuoy-C in the change in suicidal ideation severity from baseline to T1, but there were significantly greater reductions in the severity of suicide ideation for LifeBuoy+Eng relative to LifeBuoy-C from baseline to T2, and from baseline to T3 (see Supplemental Table S1b ), resulting in significantly lower suicidal ideation scores for LifeBuoy+Eng relative to LifeBuoy-C at T2 (B = -6∙55, 95% CI [-8∙95, -4∙15], t[1610∙58] = -5∙35, p < ∙001, d = 0∙57) and T3 (B = -3∙17, 95% CI [-5∙62, -0∙72], t[1659∙39] = -2∙54, p = ∙011, d = 0∙26).

*PHQ9*

There were no significant differences between LifeBuoy and LifeBuoy-C in the change in depression level from baseline to T1, or from baseline to T2, but there was a significantly greater reduction in depression level for LifeBuoy relative to LifeBuoy-C from baseline to T3 (see Supplemental Table S1a), resulting in significantly lower depression scores for LifeBuoy relative to LifeBuoy-C at T3 (B = -1∙42, 95% CI [-2∙69, -0∙15], t[1648∙76] = -2∙20, p = ∙028, d = 0∙22). A similar pattern of results was found when comparing LifeBuoy+Eng with LifeBuoy-C, but with earlier gains for the LifeBuoy+Eng condition. There was no significant difference between LifeBuoy+Eng and LifeBuoy-C in the change in depression level from baseline to T1, but there were significantly greater reductions in depression level for LifeBuoy+Eng relative to LifeBuoy-C from baseline to T2, and from baseline to T3 (see Supplemental Table S1b), resulting in significantly lower depression scores for LifeBuoy+Eng relative to LifeBuoy-C at T2 (B = -1∙46, 95% CI [-2∙71, -0∙21], t[1607∙69] = -2∙29, p = ∙022, d = 0∙24) and T3 (B = -1∙33, 95% CI [-2∙60, -0∙06], t[1650∙12] = -2∙05, p = ∙041, d = 0∙22).

*GAD7*

Generalised anxiety levels significantly improved from baseline to T1, T2, and T3 for all three conditions (Bs ranged from -2∙75 to -0∙87, all |ts| > 2∙48, all ps <∙013, all ds ranged from 0∙18 to 0∙58). However, there were no differential Time x Condition effects when comparing LifeBuoy and LifeBuoy-C (see Supplemental Table S1a) and when comparing LifeBuoy+Eng and LifeBuoy-C (see Supplemental Table S1b).

*Suicide attempts*

There were no significant changes in the odds of participants who attempted suicide in the last 30 days from baseline to T1, T2 or T3 for any of the three conditions (ORs ranged from 0∙23 to 3∙10, all |zs| < 1∙92, all ps > ∙054), with no differential Time x Condition effects when comparing LifeBuoy and LifeBuoy-C (see S1a_Table) and when comparing LifeBuoy+Eng and LifeBuoy-C (see Supplemental Table S1b).

*Non-suicidal self-harm*

There were no significant changes in the odds of participants who engaged in NSSH in the last 30 days from baseline to T1 and T2 for any of the three conditions (ORs ranged from 0∙57 to 1∙45, all |zs| < 1∙83, all ps > ∙067), with no differential Time x Condition effects when comparing LifeBuoy and LifeBuoy-C (see Supplemental Table S1a) and when comparing LifeBuoy+Eng and LifeBuoy-C (see Supplemental Table S1b). At T3, all three conditions had significantly reduced odds of participants who engaged in NSSH in the last 30 days relative to baseline (ORs ranged from 0∙43 to 0∙53, both |zs| > 2∙05, all ps <∙041), but there were no differential Time x Condition effects when comparing LifeBuoy and LifeBuoy-C (see Supplemental Table S1a) and when comparing LifeBuoy+Eng and LifeBuoy-C (see Supplemental Table S1b).

# Supplemental Table S1a. Model-based estimates and standard error for primary and secondary outcomes at each time point for LifeBuoy vs LifeBuoy-C

|  | *LifeBuoy* | | | | *LifeBuoy-C (sham) control* | | | | 30-day assessment (T1) | | | 60-day assessment (T2) | | | 120-day assessment (T3) | | |
| --- | --- | --- | --- | --- | --- | --- | --- | --- | --- | --- | --- | --- | --- | --- | --- | --- | --- |
|  |  |  |  |  |  |  |  |  | Time x Condition | | | Time x Condition | | | Time x Condition | | |
|  | T0 | T1 | T2 | T3 | T0 | T1 | T2 | T3 | *statistic* | *df* | *p* | *statistic* | *df* | *p* | *statistic* | *df* | *p* |
|  | (n=230) | (n=158) | (n=139) | (n=129) | (n=233) | (n=160) | (n=143) | (n=137) |  |  |  |  |  |  |  |  |  |
| *Primary outcome* | | | | | | | | | | | | | | | | | |
| Ideation  SIDAS, M (SE) | 25∙86 (0∙73) | 20∙54 (0∙83) | 17∙72 (0∙87) | 15∙88 (0∙89) | 26∙67 (0∙73) | 21∙34 (0∙83) | 22∙56  (0∙86) | 20∙01 (0∙87) | t=0∙01 | 1422∙47 | ∙995 | t=-3∙47 | 1431∙42 | < ∙001 | t=-3∙72 | 1471∙29 | < ∙001 |
| *Secondary outcomes* | | | | | | | | | | | | | | | | | |
| Depression PHQ-9,  M (SE) | 18∙47 (0∙38) | 16∙15 (0∙43) | 15∙92 (0∙45) | 14∙34 (0∙46) | 18∙31 (0∙38) | 16∙03 (0∙43) | 16∙21 (0∙45) | 15∙76 (0∙45) | t=-0∙06 | 1422∙90 | ∙956 | t=-0∙73 | 1431∙36 | ∙464 | t=-1∙98 | 1470∙13 | ∙048 |
| Anxiety  GAD-7,  M (SE) | 13∙27 (0∙34) | 12∙40 (0∙38) | 12∙05 (0∙40) | 10∙73 (0∙41) | 13∙52 (0∙34) | 11∙85 (0∙38) | 11∙69 (0∙39) | 11∙29 (0∙40) | t=1∙59 | 1406∙07 | ∙112 | t=1∙17 | 1413∙90 | ∙243 | t=0∙33 | 1452∙02 | ∙741 |
| Suicide attempts in past 30 days, n (%) | 18 (7∙8) | 14 (8∙9) | 10 (7∙2) | 5 (3∙9) | 13 (5∙6) | 15 (9∙4) | 12 (8∙4) | 7 (5∙1) | z=-0∙92 | - | ∙356 | z=-1∙12 | - | ∙264 | z=-1∙47 | - | ∙141 |
| NSSH in past 30 days, n (%) | 110 (47∙8) | 67 (42∙4) | 54 (38∙8) | 45 (34∙9) | 109 (46∙8) | 72 (45∙0) | 60 (42∙0) | 46 (33∙6) | z=-0∙56 | - | ∙577 | z=-0∙61 | - | ∙539 | z=-0∙33 | - | ∙743 |

Note. Time x Condition interactions reflect the period from baseline to the specified timepoint. SIDAS – Suicidal Ideation Attributes Scale; PHQ-9 – Patient Health Questionnaire 9; GAD-7 – Generalised Anxiety Disorder-7; NSSH – Non-Suicidal Self-Harm.

# Supplemental Table S1b. Model-based estimates and standard error for primary and secondary outcomes at each time point for LifeBuoy+Eng vs LifeBuoy-C

|  | *LifeBuoy+Eng* | | | | *LifeBuoy-C (sham) control* | | | | 30-day assessment (T1) | | | 60-day assessment (T2) | | | 120-day assessment (T3) | | |
| --- | --- | --- | --- | --- | --- | --- | --- | --- | --- | --- | --- | --- | --- | --- | --- | --- | --- |
|  |  |  |  |  |  |  |  |  | Time x Condition | | | Time x Condition | | | Time x Condition | | |
|  | T0 | T1 | T2 | T3 | T0 | T1 | T2 | T3 | *statistic* | *df* | *p* | *statistic* | *df* | *p* | *statistic* | *df* | *p* |
|  | (n=229) | (n=139) | (n=138) | (n=127) | (n=233) | (n=160) | (n=143) | (n=137) |  |  |  |  |  |  |  |  |  |
| *Primary outcome* | | | | | | | | | | | | | | | | | |
| Ideation  SIDAS, M (SE) | 26∙45 (0∙73) | 20∙81 (0∙87) | 16∙02 (0∙87) | 16∙84 (0∙90) | 26∙67 (0∙73) | 21∙34 (0∙83) | 22∙56  (0∙86) | 20∙01 (0∙87) | t=-0∙27 | 1436∙68 | ∙790 | t=-5∙42 | 1440∙56 | < ∙001 | t=-4∙67 | 1483∙07 | < ∙001 |
| *Secondary outcomes* | | | | | | | | | | | | | | | | | |
| Depression PHQ-9,  M (SE) | 18∙62 (0∙38) | 15∙75 (0∙45) | 14∙74 (0∙46) | 14∙43 (0∙47) | 18∙31 (0∙38) | 16∙03 (0∙43) | 16∙21 (0∙45) | 15∙76 (0∙45) | t=-0∙99 | 1438∙30 | ∙322 | t=-2∙93 | 1442∙41 | ∙004 | t=-3∙32 | 1484∙97 | < ∙001 |
| Anxiety  GAD-7,  M (SE) | 13∙22 (0∙34) | 11∙36 (0∙40) | 10∙84 (0∙41) | 10∙47 (0∙41) | 13∙52 (0∙34) | 11∙85 (0∙38) | 11∙69 (0∙39) | 11∙29 (0∙40) | t=-0∙39 | 1420∙96 | ∙698 | t=-1∙05 | 1425∙25 | ∙295 | t=-1∙20 | 1467∙29 | ∙231 |
| Suicide attempts in past 30 days, n (%) | 17 (7∙4) | 11 (7∙9) | 10 (7∙2) | 8 (6∙3) | 13 (5∙6) | 15 (9∙4) | 12 (8∙4) | 7 (5∙1) | z=-1∙09 | - | ∙276 | z=-1∙24 | - | ∙217 | z=-0∙95 | - | ∙341 |
| NSSH in past 30 days, n (%) | 105 (45∙9) | 72 (51∙8) | 63 (45∙7) | 46 (36∙2) | 109 (46∙8) | 72 (45∙0) | 60 (42∙0) | 46 (33∙6) | z=1∙16 | - | ∙246 | z=0∙65 | - | ∙519 | z=0∙65 | - | ∙515 |

Note. Time x Condition interactions reflect the period from baseline to the specified timepoint. SIDAS – Suicidal Ideation Attributes Scale; PHQ-9 – Patient Health Questionnaire 9; GAD-7 – Generalised Anxiety Disorder-7; NSSH – Non-Suicidal Self-Harm.

# Supplemental Appendix 2

# Analyses of primary outcome comparing the two intervention conditions: LifeBuoy to LifeBuoy+Eng

There were no differential Time x Condition effects when comparing LifeBuoy+Eng with LifeBuoy on suicidal ideation severity at any time point (see S2_Table). There were no significant differences in suicidal ideation scores between LifeBuoy+Eng and LifeBuoy at T1 (B = 0∙28, 95% CI [-2∙09, 2∙65], t[1559∙46] = 0∙23, p = ∙818, d = 0∙02), T2 (B = -1∙70, 95% CI [-4∙12, 0∙71], t[1615∙17] = -1∙38, p = ∙167, d = -0∙15), or T3 (B = 0∙96, 95% CI [-1∙52, 3∙44], t[1678∙96] = 0∙76, p = ∙447, d = 0∙08)

# Supplemental Table S2. Model-based estimates and standard error for primary outcome at each time point for LifeBuoy+Eng vs LifeBuoy

|  | *LifeBuoy+Eng* | | | | *LifeBuoy* | | | | 30-day assessment (T1) | | | 60-day assessment (T2) | | | 120-day assessment (T3) | | |
| --- | --- | --- | --- | --- | --- | --- | --- | --- | --- | --- | --- | --- | --- | --- | --- | --- | --- |
|  |  |  |  |  |  |  |  |  | Time x Condition | | | Time x Condition | | | Time x Condition | | |
|  | T0 | T1 | T2 | T3 | T0 | T1 | T2 | T3 | *statistic* | *df* | *p* | *statistic* | *df* | *p* | *statistic* | *df* | *p* |
|  | (n=229) | (n=139) | (n=138) | (n=127) | (n=230) | (n=158) | (n=139) | (n=129) |  |  |  |  |  |  |  |  |  |
| *Primary outcome* | | | | | | | | | | | | | | | | | |
| Suicidal ideation, M (SE) | 26∙45 (0∙73) | 20∙81 (0∙87) | 16∙02 (0∙87) | 16∙84 (0∙90) | 25∙86 (0∙73) | 20∙54 (0∙83) | 17∙72 (0∙87) | 15∙88 (0∙89) | t=-0∙27 | 1441∙78 | ∙787 | t=-1∙95 | 1446∙27 | ∙051 | t=-0∙96 | 1491∙76 | ∙338 |

# Supplemental Table S3. Serious adverse events x condition

|  | **LifeBuoy (n=230)** | **LifeBuoy+Eng (n=229)** | **LifeBuoy-C (n=233)** |
| --- | --- | --- | --- |
| Total SAEs during trial | 15 | 17 | 50 |
| No. unique participants reporting SAEs | 8 | 12 | 22 |
| **T1 (30-day assessment)** | | | |
| No. hospital presenting suicide attempts | 3 | 3 | 12 |
| No. hospital presenting NSSH incidents | 3 | 1 | 8 |
| **T2 (60-day assessment)** | | | |
| No. hospital presenting suicide attempts | 3 | 4 | 9 |
| No. hospital presenting NSSH incidents | 2 | 0 | 7 |
| **T3 (120-day assessment)** | | | |
| No. hospital presenting suicide attempts | 1 | 4 | 6 |
| No. hospital presenting NSSH incidents | 3 | 5 | 8 |

SAE=Serious Adverse Event (hospital presenting non-suicidal self -harm and/or suicide attempt); NSSH=non-suicidal self-harm; No.=number

# Supplemental Appendix 3

# Sensitivity analysis – primary analysis adjusting for variables associated with attrition

Adjusting for variables associated with attrition, there were no significant differences between the intervention conditions combined and LifeBuoy-C in the change in suicidal ideation severity from baseline to T1, but there were significantly greater reductions in the severity of suicide ideation in the two intervention conditions combined relative to LifeBuoy-C from baseline to T2, and from baseline to T3 (see Supplemental Table S2b). This resulted in significantly lower suicidal ideation scores for the two intervention conditions combined relative to LifeBuoy-C at T2 (B = -5∙72, 95% CI [-7∙76, -3∙68], t[1638∙66] = -5∙50, p < ∙001, d = 0∙48) and T3 (B = -3∙68, 95% CI [-5∙76, -1∙60], t[1683∙55] = -3∙47, p < ∙001, d = 0∙30

# Supplemental Table S4a. Variables associated with survey attrition (survey attrition sensitivity analysis)

|  | **T1 survey attrition** | |  | **T2 survey attrition** | |  | **T3 survey attrition** | |  |
| --- | --- | --- | --- | --- | --- | --- | --- | --- | --- |
|  | Survey loss  (n=235) | Survey completers  (n=457) | *p*s | Survey loss  (n=272) | Survey completers (n=420) | *p*s | Survey loss  (n=299) | Survey completers (n=393) | *p*s |
| Gender Identity (n, %) |  | | | | | | | | |
| *Female* | 167 (71∙7) | 314 (68∙7) | ∙910 | 195 (71∙7) | 286 (68∙1) | ∙779 | 213 (71∙2) | 268 (68∙2) | ∙690 |
| *Male* | 33 (14∙0) | 66 (14∙4) |  | 36 (13∙2) | 63 (15∙0) |  | 43 (14∙4) | 56 (14∙2) |  |
| *Non-binary* | 30 (12∙8) | 67 (14∙7) |  | 35 (12∙9) | 62 (14∙8) |  | 38 (12∙7) | 59 (15∙0) |  |
| *Prefer not to answer* | 5 (2∙1) | 10 (2∙2) |  | 6 (2∙2) | 9 (2∙1) |  | 5 (1∙7) | 10 (2∙5) |  |
| *Gender assigned at birth (n, %)* |  |  |  |  |  |  |  |  |  |
| *Female* | 207 (88∙5) | 398 (87∙3) | ∙655 | 237 (87∙5) | 368 (87∙8) | ∙884 | 263 (88∙3) | 342 (87∙2) | ∙689 |
| *Male* | 27 (11∙5) | 58 (12∙7) |  | 34 (12∙5) | 51 (12∙2) |  | 35 (11∙7) | 50 (12∙8) |  |
| Age (M, SD) | 20∙09 (2∙45) | 19∙82 (2∙53) | ∙178 | 20∙15 (2∙50) | 19∙76 (2∙53) | **∙050** | 20∙06 (2∙46) | 19∙80 (2∙57) | ∙192 |
| Identifies as LGBQ+, n (%) | 128 (54∙5) | 272 (59∙5) | ∙203 | 159 (58∙5) | 241 (57∙4) | ∙780 | 167 (55∙9) | 233 (59∙3) | ∙365 |
| Education (n, %) |  | | | | | | | | |
| *High school qualification* | 139 (59∙1) | 285 (62∙4) | ∙708 | 164 (60∙3) | 260 (61∙9) | ∙890 | 178 (59∙5) | 246 (62∙6) | ∙219 |
| *Graduate certificate or diploma* | 59 (25∙1) | 107 (23∙4) |  | 66 (24∙3) | 100 (23∙8) |  | 81 (27∙1) | 85 (21∙6) |  |
| *University Degree* | 37 (15∙7) | 65 (14∙2) |  | 42 (15∙4) | 60 (14∙3) |  | 40 (13∙4) | 62 (15∙8) |  |
| Living Arrangements (n, %) |  | | | | | | | | |
| *With parents or other family* | 136 (57∙9) | 292 (63∙9) | ∙088 | 162 (59∙6) | 266 (63∙3) | ∙303 | 177 (59∙2) | 251 (63∙9) | ∙078 |
| *With partner, friends, or flatmates* | 80 (30∙4) | 115 (25∙2) |  | 85 (31∙3) | 110 (26∙2) |  | 97 (32∙4) | 98 (24∙9) |  |
| *Alone* | 13 (5∙5) | 33 (7∙2) |  | 19 (7∙0) | 27 (6∙4) |  | 19 (6∙4) | 27 (6∙9) |  |
| *Other* | 6 (2∙6) | 17 (3∙7) |  | 6 (2∙2) | 17 (4∙0) |  | 6 (2∙0) | 17 (4∙3) |  |
| Not in intimate relationship, n (%) | 140 (59∙6) | 287 (62∙8) | ∙408 | 159 (58∙5) | 268 (63∙8) | ∙157 | 176 (58∙9) | 251 (63∙9) | ∙180 |
| Lives in metropolitan area, n (%) | 189 (80∙4) | 373 (81∙6) | ∙703 | 221 (81∙3) | 341 (81∙2) | ∙984 | 243 (81∙3) | 319 (81∙2) | ∙973 |
| English language only speaker, n (%) | 199 (84∙7) | 363 (79∙4) | ∙094 | 231 (84∙9) | 331 (78∙8) | **∙044** | 253 (84∙6) | 309 (78∙6) | **∙046** |
| Employment status, n (%) |  | | | | | | | | |
| *Unemployed* | 35 (14∙9) | 49 (10∙7) | **∙002** | 41 (15∙1) | 43 (10∙2) | **∙002** | 42 (14∙0) | 42 (10∙7) | ∙131 |
| *Student (school, university)* | 99 (42∙1) | 256 (56∙0) |  | 117 (43∙0) | 238 (56∙7) |  | 141 (47∙2) | 214 (54∙5) |  |
| *Paid employment*  *(casual/part-/full-time)* | 101 (43∙0) | 152 (33∙3) |  | 114 (41∙9) | 139 (33∙1) |  | 116 (38∙8) | 137 (34∙9) |  |
| History of mental ill-health, yes (n %) | 226 (96∙2) | 436 (95∙4) | ∙640 | 260 (95∙6) | 402 (95∙7) | ∙937 | 287 (96∙0) | 375 (95∙4) | ∙717 |
| Ever seen a mental health professional, n (%) | 199 (84∙7) | 386 (84∙5) | ∙940 | 233 (85∙7) | 352 (83∙8) | ∙510 | 258 (86∙3) | 327 (83∙2) | ∙267 |
| Currently receiving mental health treatment (pharmaco- and/or psychotherapy), n (%) | 142 (60∙4) | 294 (64∙3) | ∙313 | 166 (61∙0) | 270 (64∙3) | ∙386 | 182 (60∙9) | 254 (64∙6) | ∙310 |
| Age onset of suicidal ideation (M, SD) | 13∙56 (3∙19) | 13∙36 (3∙05) | ∙419 | 13∙46 (3∙16) | 13∙41 (3∙05) | ∙816 | 13∙35 (3∙07) | 13∙49 (3∙11) | ∙546 |
| Trait impulsivity scores (M, SD) | 20∙32 (4∙75) | 19∙51 (4∙57) | **∙029** | 20∙32 (4∙66) | 19∙43 (4∙61) | **∙014** | 20∙45 (4∙52) | 19∙27 (4∙68) | **∙001** |
| SIDAS scores (M, SD) | 26∙99 (9∙95) | 25∙99 (10∙02) | ∙212 | 27∙06 (10∙01) | 25∙86 (9∙98) | ∙123 | 26∙48 (10∙00) | 26∙21 (10∙01) | ∙721 |
| PHQ-9 scores (M, SD) | 18∙72 (4∙97) | 18∙33 (5∙49) | ∙360 | 18∙88 (5∙04) | 18∙20 (5∙48) | ∙097 | 18∙75 (5∙10) | 18∙25 (5∙48) | ∙221 |
| GAD-7 scores (M, SD) | 13∙35 (4∙72) | 13∙33 (4∙92) | ∙971 | 13∙61 (4∙71) | 13∙16 (4∙94) | ∙238 | 13∙66 (4∙64) | 13∙10 (4∙99) | ∙131 |
| Suicide attempt in past 30 days, n (%) | 17 (7∙2) | 35 (7∙7) | ∙841 | 16 (5∙9) | 36 (8∙6) | ∙190 | 20 (6∙7) | 32 (8∙1) | ∙472 |
| NSSH in past 30 days, n (%) | 109 (46∙4) | 216 (47∙3) | ∙826 | 125 (46∙0) | 200 (47∙6) | ∙669 | 139 (46∙5) | 186 (47∙3) | ∙826 |
| Lifetime suicide attempt, n (%) | 150 (63∙8) | 262 (57∙3) | ∙099 | 172 (63∙2) | 240 (57∙1) | ∙111 | 188 (62∙9) | 224 (57∙0) | ∙119 |
| Lifetime NSSH, n (%) | 211 (89∙8) | 408 (89∙3) | ∙836 | 246 (90∙4) | 373 (88∙8) | ∙495 | 274 (91∙6) | 345 (87∙8) | ∙102 |

# Supplemental Table S4b. Sensitivity analysis – primary analysis adjusting for variables associated with attrition

|  | LifeBuoy and LifeBuoy+Eng interventions combined | | | | | *LifeBuoy-C (sham) control* | | | | 30-day assessment (T1) | | | 60-day assessment (T2) | | | 120-day assessment (T3) | | |
| --- | --- | --- | --- | --- | --- | --- | --- | --- | --- | --- | --- | --- | --- | --- | --- | --- | --- | --- |
|  |  |  |  |  |  |  |  |  |  | Time x Condition | | | Time x Condition | | | Time x Condition | | |
|  | T0 | T1 | | T2 | T3 | T0 | T1 | T2 | T3 | *statistic* | *df* | *p* | *statistic* | *df* | *p* | *statistic* | *df* | *p* |
|  | (n=459) | (n=297) | | (n=277) | (n=256) | (n=233) | (n=160) | (n=143) | (n=137) |  |  |  |  |  |  |  |  |  |
| *Primary outcome* | | | | | | | | | | | | | | | | | | |
| Suicidal ideation, M (SE) | 25∙48 (0∙65) | 20∙05 (0∙72) | 16∙26 (0∙73) | | 15∙77 (0∙75) | 26∙02 (0∙81) | 20∙77 (0∙89) | 21∙99  (0∙92) | 19∙45 (0∙93) | t=-0∙19 | 1436∙55 | ∙853 | t=-5∙16 | 1444∙43 | < ∙001 | t=-4∙88 | 1484∙94 | < ∙001 |
