# Supplementary material for: Adherence and efficacy outcomes in young Australians with suicidal ideation using a self-management app and digital engagement strategy compared with a sham app: a three-arm randomised controlled trial
Source: eClinicalMedicine. 2024 Dec 6;79:102963. doi: 10.1016/j.eclinm.2024.102963 (PMC11665678; doi:10.1016/j.eclinm.2024.102963)
Supplement: Supplemental Data S1 Protocol [file mmc2.docx]

**S1_Protocol**

**UNSW Clinical Trial Protocol**

**Physiological, Psychological, Psychiatric, Surgical or Health Interventions**

A randomised controlled trial of an m-health app and digital engagement strategy for improving treatment adherence and reducing suicidal ideation.

Final approved version, 1 March 2022

UTN: U1111-1268-2852; ANZCTR trial No.: ACTRN12621001247864

Dr Michelle Tye, UNSW Coordinating Principal Investigator

# **General Information**

| **Protocol Title** | | | | | | | |
| --- | --- | --- | --- | --- | --- | --- | --- |
| A randomised controlled trial of an m-health app and digital engagement strategy for improving treatment adherence and reducing suicidal ideation. | | | | | | | |
| **Protocol identifying number** | HC210400 | | | | | | |
| **Version Number** | 1.4 | | | **Version date** | 15 February 2022 | | |
| **Amendment History** | | | | | | | |
| **Version Number** | 1.0 | | | **Version date** | 24 May 2021 | | |
|  | 1.1 | | |  | 15 July 2021 | | |
|  | 1.2 | | |  | 26 July 2021 | | |
|  | 1.3 | | |  | 22 Oct 2021 | | |
| **Clinical Trial Sponsor** | | | | | | | |
| **Sponsor Name** | University of New South Wales | | | | | | |
| **Sponsor Contact** | Dr Ted Rohr, UNSW Sponsor's Delegate | | | | | | |
| **Telephone** | 0417844054 | | | | | | |
| **Email** | humanethics@unsw.edu.au | | | | | | |
| **Address** | UNSW Research Ethics and Compliance Support, The University of New South Wales, Sydney NSW 2052 Australia. | | | | | | |
| **Coordinating Principal Investigator** | | | | | | | |
| **Name** | Dr Michelle Tye | | | | | | |
| **Telephone** | 0415 677 477 | | | | | | |
| **Email** | michelle.torok@blackdog.org.au | | | | | | |
| **Type of Appointment with UNSW** | UNSW Employee  UNSW Conjoint  Other (Please describe) | | | | | | |
| **Principal Investigator - 1** | | | | | | | |
| **Name** | Dr Michelle Tye | | | | | | |
| **Contact** | **Email** | michelle.torok@blackdog.org.au | | | | **Telephone** | 0415677477 |
| **Site** | Black Dog Institute, Hospital Rd, Randwick NSW 2031 | | | | | | |
| **Principal Investigator - 2** | | | | | | | |
| **Name** | N/A | | | | | | |
| **Contact** | **Email** |  | | | | **Telephone** |  |
| **Site** |  | | | | | | |
| **Personnel authorised to sign the protocol and the protocol amendment(s) for the Sponsor** (ICH GCP 6.1.3) | | | | | | | |
| **Name** | Dr Michelle Tye | | | | | | |
| **Telephone** | 0415 677 477 | | | | | | |
| **Email** | michelle.torok@blackdog.org.au | | | | | | |
| **Address** | Black Dog Institute, Hospital Rd, Randwick NSW 2031 | | | | | | |
| **Human Research Ethics Committee** | | | | | | | |
| **Name** | | | UNSW HREC Committee A | | | | |
| **Status of ethical review** | | | **Approved**  **In progress**  **To be submitted** | | | | |
| **Trial Sites** | | | N/A – online in Australia | | | | |
| **Funding for the Clinical Trial** | | | | | | | |
| **Funding Body Name** | | | Philanthropic grants (Roth Foundation, Goodman Foundation, and Matana) and Australian Rotary Health [ARH]. | | | | |
| **Amount of Funding** | | | $76,080 (ARH) + $81,000 (philanthropy) | | | | |
| **Interests that the funding body has in the clinical trial** | | | The funders have a targeted interest in understanding what interventions help to reduce suicide risk in young people, however, none have any actual role in the trial conduct. | | | | |
| **Insurance for Clinical Trial** | | | | | | | |
| **Insurer** | | | **UNSW** | | | | |
| **Type of Insurance** | | | Clinical trial insurance was obtained via UNSW and a Certificate of Currency is provided (Appendix I). | | | | |
| **Confirmation of Insurance** | | | **Attached**  **In progress**  **To be submitted** | | | | |

# **Safety and Monitoring Contacts**

| **Clinical Trials Involving Physiological, Psychological, Psychiatric or Surgical Interventions** | |
| --- | --- |
| **Qualified Physician/Medical Expert** | |
| **Name** | Professor Samuel Harvey |
| **Telephone** |  |
| **Email** | s.harvey@unsw.edu.au |
| **Address** | Black Dog Institute, Hospital Rd, Randwick NSW 2031 |
| **Sponsors Independent Physician/Medical Expert** | |
| **Name** | Professor Jennie Hudson |
| **Telephone** |  |
| **Email** | jennie.hudson@blackdog.org.au |
| **Address** | Black Dog Institute, Hospital Rd, Randwick NSW 2031 |
| **Independent Safety Monitoring Board or Data Safety Monitoring Board Members** | |
| - List the members of the safety monitoring board. - Members with relevant expertise in clinical trials and the subject matter area will be invited after ethics approval has been received, and before recruitment commences. | |
| **Trial Management Group** | |
| - Dr Michelle Tye - Dr Lauren McGillivray - Dr Quincy Wong - Dr Jin Han - Mr Daniel Gan - Assoc Prof Sarah Hetrick | |

# **Delegation of Clinical Trial Duties**

Responsibilities for the conduct and oversight for the trial are delegated to you as the Coordinating Principal Investigator. You may delegate trial related responsibilities to the listed Principal Investigator(s) and any trial-related personnel. All trial-related duties delegated by the Coordinating Principal Investigator or Principal Investigator(s) and trial-related personnel must only be delegated to those that are qualified by experience and training. Delegated responsibilities must be retained in the [UNSW Clinical Trial Delegation Log](https://research.unsw.edu.au/document/Clinical%20Trial%20Delegations%20Log.docx) (Appendix A). The UNSW Sponsor's Delegate is to be notified of the following:

- Protocol deviation reports outlined in the UNSW Research Misconduct Procedure.
- Any serious breach of Good Clinical Practice, the clinical trial protocol, the clinical trial standard operating procedures, or the human ethics approval that is likely to affect to a significant degree the safety or rights of participants or the reliability and robustness of the data generated in the clinical trial.
- Significant safety issues that are likely to (or have the potential to) affect to a significant degree the safety or rights of participants or the reliability and robustness of the data generated in the clinical trial.
- Urgent safety measures implemented to remove or prevent a significant safety issue.
- Safety reports relating to the continuation, suspension, or discontinuation of the clinical trial for safety reasons.
- Non-compliance with the protocol, SOPs, GCP, and applicable regulatory requirement(s) significantly affects or can potentially affect human subject protection or reliability of trial results significantly.
- Participant complaints or concerns received concerning the conduct of the research.
- Significant modifications to the clinical trial are likely to affect a significant degree the safety or rights of participants or the reliability and robustness of the data generated in the clinical trial.
- Addition of participating trial sites, contractual arrangements at participating sites or modifications to legal agreements.
- The intention to conduct the trial in other countries.

# **Trial Objectives and Purpose**

The LifeBuoy study has been designed to overcome challenges in: (i) the availability of effective digital health interventions for young people specifically for suicidal ideation AND (ii) how to promote engagement with such products to optimise therapeutic benefits. We plan to address these challenges by conducting a large 3-arm parallel randomised controlled trial (RCT) to test whether suicidal ideation can be reduced - and intervention engagement increased - using a therapeutic intervention delivered by smartphone application alongside a multicomponent digital engagement strategy.

Primary outcomes

Efficacy of the lifebuoy app: One primary objective of the current trial is to evaluate the efficacy of a mHealth application — LifeBuoy — for reducing suicidal ideation over time among young people relative to a control (non-therapeutic) app in a randomised controlled trial (RCT). This trial will answer questions that are important to making decisions about how (and whether) to scale up these interventions. More specifically, comparing 2-arms (both interventions combined vs. control arm), we will assess the efficacy of the LifeBuoy app in reducing suicide ideation among young people, as measured by the Suicidal Ideation Attributes Scale (SIDAS; [9]), relative to a placebo attention control condition. Changes in suicidal ideation will be assessed by comparing the intervention to the attention placebo control condition at 0-days (baseline, T0), 30-days (T1), 60-days (T2), and 120-days (T3).

Efficacy of the engagement strategy: the other primary objective is to explore whether participants who receive LifeBuoy + a digital engagement strategy report higher rates of app usage (measured in terms of the number of modules completed, number of app logins, and total time spent on the app) relative to the LifeBuoy-only condition. This trial will answer important questions about whether, and what, digital strategies improve user engagement, addressing key knowledge gaps in the digital health field. Previous research has shown that technology-based strategies can potentially improve user engagement [14]. However, a plausible reason why many such strategies fail is because they do not sufficiently address the needs of users and are thus not well-received [15]. Moreover, few studies have examined the efficacy of social media (e.g., Instagram) and blog posts in promoting greater engagement with DMHIs. More specifically, we will examine the efficacy of a digital engagement strategy – designed to optimise use of the LifeBuoy app – on app adherence (measured in the following ways: number of modules / activities / exercises completed; number and frequency of app log-ins; total time spent on the app) by comparing 2-arms: LifeBuoy + engagement strategy versus LifeBuoy-only at 0-days (baseline, T0), 30-days (T1), 60-days (T2), and 120-days (T3).

Secondary outcomes

1. To determine whether the LifeBuoy app reduces incidents of suicide attempt and non-suicidal self injury relative to the control condition at T1, T2, and T3.
2. To assess whether the LifeBuoy app reduces depression and anxiety symptoms relative to the control condition at T1, T2, and T3.
3. To examine whether the LifeBuoy app + engagement condition reduces suicidal ideation relative to the LifeBuoy-only condition at T1, T2, and T3.

# **Background Information**

Youth suicidal ideation and behaviour is a worrying public health issue due to its widespread prevalence and grave consequences. In Australia, suicide is the leading cause of death among individuals between 15 to 44 years of age [1]. In addition, suicidal ideation is relatively common among youth in the general population, with 12-month prevalence estimates as high as 24% in those aged 18 or younger [2]. Over one‐third of adolescents who experience suicidal ideation go on to attempt suicide [3]. Furthermore, it has been estimated that youth suicide carries significant economic costs, estimated to be $511M per annum [4]. High suicide rates among youth are exacerbated by their reluctance to seek help for psychological distress and suicidality. A recent review [5] reported that of 12,006 individuals with past-year suicide ideation, plans, and/or attempts, a weighted average of less than 30% sought help from mental health services. Lower rates of help-seeking were associated with being younger, being male, and cultural influences [5,6]. Potentially up to 70% of young people have not attempted or received access to mental health services. Traditional face-to face treatment services also have numerous economic, logistical, and personal barriers (i.e., stigma) that prevent most young Australians from accessing them [7].

Digital Mental Health interventions (DMHIs)—psychotherapeutic interventions delivered using digital (web- or smartphone-based) platforms—offer a new opportunity to improve access to support and provide high fidelity/cost-effective treatment, thereby addressing gaps observed in current population and health system approaches [8]. Importantly, recent meta-analytic evidence supports the effectiveness of DMHIs in mitigating suicidal ideation [9].

However, two significant issues warrant further investigation. At present, few digital interventions targeting suicidal ideation have been developed and rigorously tested, and none specifically for young people. This is despite estimates that 94% of young people own a smartphone and that they spend up to one-third of their day using electronic devices (phones, computers) [9]. Considering the levels of smartphone ownership and use, it is surprising that DMHIs remain relatively unexplored as a health solution in suicide prevention. Furthermore, the adoption of DMHIs in routine practice has been poor, in part, because of suboptimal rates of user engagement with these interventions [10-11]. Recent meta-analytic findings report that over 75% of users do not complete these interventions [12], and that attrition is especially salient among youth and young adult users [13]. Hence, there is a compelling need for the development and testing of effective strategies for enhancing user engagement with DMHIs.

To date, only one systematic review [14] has investigated the effectiveness of technology-based strategies in promoting user engagement with digital interventions (14 studies, n = 8,774). The review found that technology-based strategies—mostly in the form of prompts via email, text message or telephone—showed modest effects in promoting engagement than when no strategy was employed; however, the authors cautioned that the findings be considered in light of several limitations, including the small number of studies, small sample sizes in some of the studies, and heterogeneity in the methods employed across studies [14]. Importantly, only 8 of the 14 digital interventions discussed in the review were DMHIs. In summary, evidence of the effectiveness of engagement strategies in the context of DMHIs is scant and inconclusive.

#

# **Statement of Compliance**

The clinical trial will be conducted in compliance with the following guidelines and documentation:

- [ICH Guidelines for Good Clinical Practice (GCP)](https://www.tga.gov.au/publication/note-guidance-good-clinical-practice)
- [National Statement on Ethical Conduct in Human Research](https://nhmrc.gov.au/about-us/publications/national-statement-ethical-conduct-human-research-2007-updated-2018) (National Statement)
- As approved by the Human Research Ethics Committee (HREC), the clinical trial protocol is responsible for monitoring the trial's conduct.
- The responsibilities set out by the UNSW Sponsors Delegate.

The onsite or remote monitoring standard operating procedures as put in place by the clinical trial sponsor.

#

# **Trial Design**

This parallel design randomised controlled trial will involve three arms to answer the main objectives outlined in Section 4: 1) attentional control, 2) LifeBuoy only, and 3) LifeBuoy + engagement strategy. Participants will be followed up over four time-points: 0-days (baseline, T0), (0 days), 1-month (30- days (T1), 2-months (60- days (T2), and 4-months (120-days (T3).

The primary endpoint is change in suicidal ideation at T1 (day-30) relative to T0 (baseline, day-0).

The secondary endpoint is adherence to the LifeBuoy app across time, defined as the number of modules completed by participants at the T1, T2, and T3 post-baseline time-points. Participant module access and module completion data will be automatically collected via the LifeBuoy app.

The following flow chart (Figure 1) describes how an individual participant will progress through the phases of the research process:


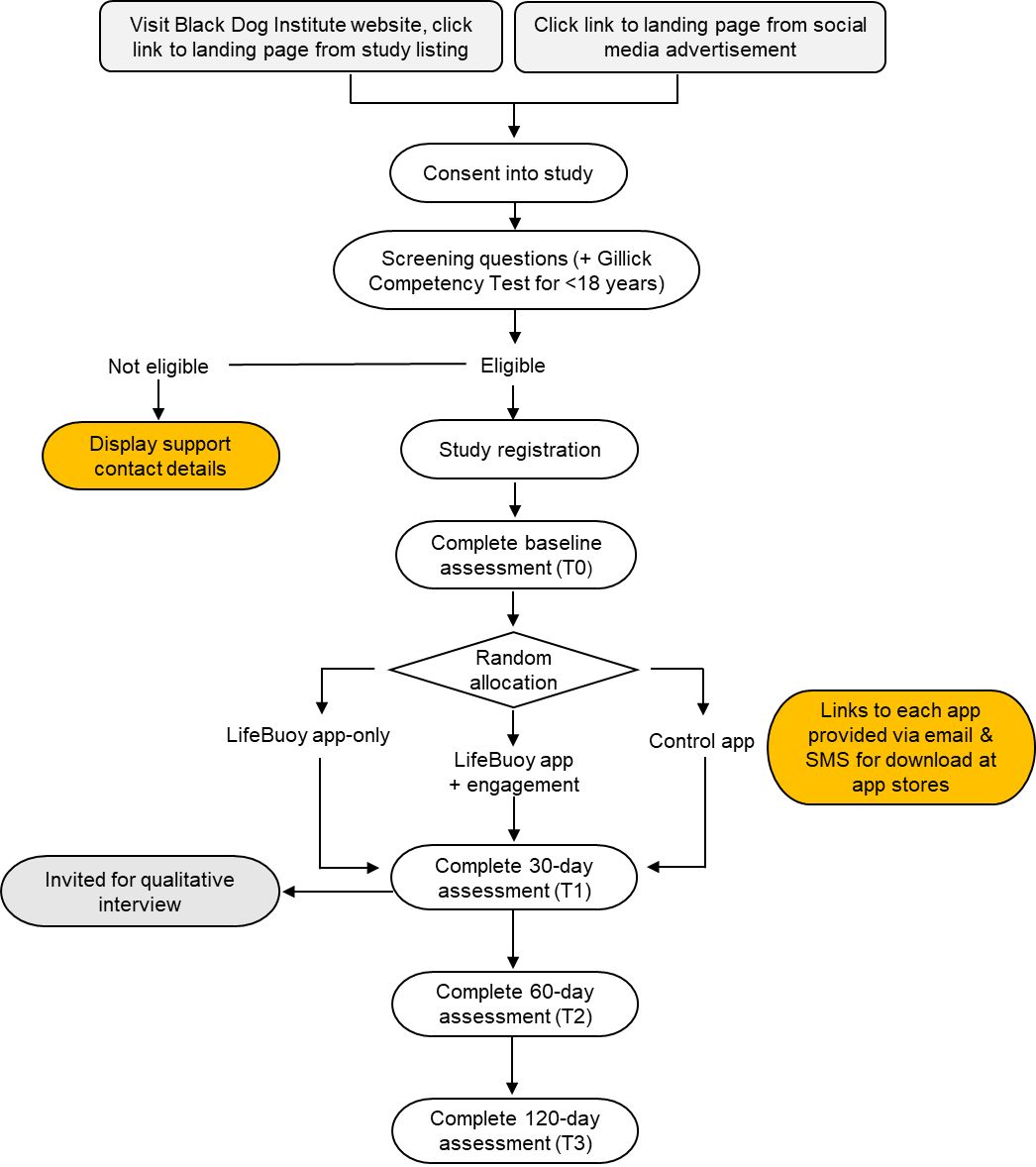


*Figure 1: Study Process Flow*

Randomisation to treatment arms will occur on a 1:1:1 ratio using a block design using an automated web-based platform tailored for this project. All study assessments will be via electronic self-report. There are no outcome assessors.

Participants will have until the end of the 120-day period to access the apps. They will be prospectively followed up over four time-points: 0-days (baseline, T0), (0 days), 30- days (T1), 60- days (T2), and 120-days post baseline (T3). After completion of the T1 assessment, participants from the LifeBuoy (n=20) and LifeBuoy + engagement strategy (n=20) conditions will be invited to participate in a qualitative interview about the acceptability of the app and engagement strategy (n=40 in total). Participants will be recruited on a first-in first-served basis, and interviews will be conducted within 1-month of completing the T1 assessment to minimise memory failure relating to the engagement strategies.

The discontinuation criteria for individual participants will include if they experience a serious adverse event (Section 9.6 details withdrawal procedures).

The following accountability procedures for the intervention and control apps will apply:

- Software version control ensuring only approved/tested version of each intervention is used in the trial.

Randomization codes will be generated and retained automatically by the Black Dog Institute’s research engine.

Responsibility for holding and securing the randomisation list and performing any code break will be assigned in the Delegations Log. The list holder cannot be the Principal Investigator, or any other staff member involved in:

- day-to-day trial operations; or
- primary data analysis.

Because individual participants will be aware of, and able to disclose, the intervention to which they were allocated, codes may be broken only in the following situations:

- On completion of the primary data analysis, certified as complete by the Principal Investigator; or
- If required by the trial Data Safety Monitoring Board (see section 13, Ethics) in writing to the Principal Investigator.

Any code break shall be recorded in the Safety Monitoring Register and reported to the Sponsor).

Serious adverse events will be reported into the Data Monitoring Safety Board (DSMB, see Appendix). The trial manager will record the number of notifications at each assessment in the Report for DSMB document and will notify the DSMB every 3-months on how many alerts were triggered, and what follow-up was carried out. They will record these in a Case Report spreadsheet which will be securely stored on the UNSW server.

# **Sample Size**

The plan is to enrol 669 subjects to this trial. Sample size calculations were based on the primary aims. For Aim 1, to detect an expected minimum effect size of d=0.45 between the LifeBuoy intervention and the control condition on the primary measure of suicidal ideation (SIDAS), determined from the prior LifeBuoy trial (paper in review), with α = 0.01, power = 0.95, and assuming a 0.50 correlation between repeated measures, 112 subjects are needed in each condition. As a three-arm trial, with a 1:1:1 allocation, this means N=336 subjects are required to detect this effect. In addition, assuming an attrition rate of 25% in each group at our 30-day assessment based on the previous trial of LifeBuoy, 450 participants are needed (150 in each arm, rounded up). This sample size is sufficient to detect the expected effects associated with primary aim 1. For Aim 2, our recent systematic review of the engagement literature yielded three studies which were similar in design and population to our study34-36. The effect sizes in these studies varied from d = .04 to .53 reflecting a difference in user engagement when comparing a digital intervention only and the same digital intervention with an engagement strategy. Given the small number of studies, there is insufficient information to assume that effect sizes are normally distributed. Thus, the median effect size among the 3 studies (d = .38) was used as a benchmark. To detect an effect size of d=0.38 for primary aim 2 (alpha 0.01, power 0.95, assuming a 0.50 correlation between repeated measures, and 25% attrition), 223 participants are needed in each arm (rounded up). With a 1:1:1 allocation, this means N=669 subjects are required. Therefore, the larger sample size of 669 participants will be chosen to meet requirements of both primary aims. Qualitative interviews with 40 young people from the LifeBuoy and LifeBuoy + engagement group (minimum of 20 participants from the latter) will be recruited at the 30-day assessment time point, in which they will be asked whether they are interested to take part in an interview about their experience of the app and engagement strategy. This sample size will be large enough to reach saturation of qualitative themes.

# **Selection and Withdrawal of Subjects**

## Inclusion Criteria

Study inclusion criteria are as follows:

- 17 - 24 years old.
- Living in Australia.
- Fluent in English.
- Own and have access to a smartphone.
- Experienced suicidal thoughts in the past 30 days.

## Exclusion Criteria

Study exclusion criteria are as follows:

- Have been diagnosed with psychosis or a bipolar disorder in the past 30 days (because of increased risk to safety in this vulnerable group).

## **Recruitment Strategy**

The research team will identify a national community-based sample of participants for this project by running targeted social media advertisements in 2-weeks blocks until the full sample is recruited on Black Dog Institute’s Facebook page (see Appendix J for advertisement content).

The marketing/communications team at BDI will post targeted recruitment advertisement on Facebook to the following groups of users for the purposes of maximising visibility to relevant users: Male/Female 17-24, Australia, Interests > Additional interests, National Suicide Prevention Lifeline Suicide prevention, Beyondblue, Headspace, Lifeline (crisis support service), R U OK Day, SANE (charity). This strategy has been successfully used in many studies at the Black Dog Institute, including a survey study of anxiety during COVID-19, which recruited >5,000 in less than two months and an RCT of a mHealth intervention in young people, which recruited >450 in four days.

This initial contact to potential participants will not involve real or perceived coercion or pressure to participate because the researchers will not have direct contact with potential participants; recruitment, consent, screening, registration, and questionnaires are all scheduled and completed online.

In the absence of a response to completing the baseline questionnaire following registration, reminder/follow-up contact with potential participants will be undertaken by:

- Reminder invitations will be emailed to participants if they fail to complete the baseline questionnaire, or subsequent post and follow-up questionnaires.
- No more than 2 reminder invitations will be used to avoid potential coercion or pressure to participate.

Potential participants can indicate their interest in participating by clicking on the recruitment advertisement and following the directions online.

In the T1 assessment point, participants allocated to the LifeBuoy and LifeBuoy + engagement strategy conditions will be asked whether they are interested in participating in an interview to know more about their experience with the app and engagement strategies used during the study. Participants who have consented (Appendix D) will be contacted by email to arrange a time to attend an online interview (either telephone or videoconference). Questions of the qualitative interview have been listed in Appendix E.

## **Screening**

To determine whether a participant is eligible to take part in the study the research team will conduct a screening process, after they provide study consent. The eligibility survey will ask participants if they meet the inclusion/exclusion criteria. Participants who are deemed ineligible will be directed to a webpage thanking them for their time. This will include relevant support contact details. Participants who are eligible will be directed undertake the baseline assessment (Appendix).

## **Consent**

Participants will be provided with the PISCF (Appendix) online, once they click on the recruitment advertisement. Participants will be asked to read the PISCF and will have sufficient time to consider their participation because there is no time restriction before they consent and register to participate. Participants will be advised to contact the researcher(s) if they have any questions. The PISCF will be available for participants to download at the time of reading and from the study landing page if they wish. Once they are comfortable providing their consent to participate, participants will be asked to digitally sign the consent form and click ‘submit’, which will all be managed online via the trial portal (hosted on Black Dog Institute’s research engine). Upon receipt of their consent, they will be automatedly directed to register in the study.

This study will include participants aged 17 years to be able to consent themselves into this study as mature minors. To ensure that these young people are fully aware of the study’s intent, terms, and consequences, they will be asked to complete a 5-item Gillick Competency Task (Appendix D). The Gillick Competency Task comprises multiple choice responses to 5 questions relating to what the study is about, age for eligibility, what the study involves, how many surveys are included, and whether the respondent feels pressured to participate (please see questions and pass/fail responses below). If they fail this task (by answering any of the questions incorrectly), they will not be able to complete the survey and will be offered a message saying they are unable to successfully consent to this study and a list of help resources for mental health and suicide.

## **Withdrawal of Consent or Participant**

Participants may be withdrawn from the study if:

- They experience a serious adverse event that:
  - is attributable to a study intervention or procedure; or
  - means they can no longer participate in the study; or
- They withdraw consent to take part.
- We will not be replacing participants that are withdrawn from the study.

The team clinical psychologist will follow-up with subjects withdrawn due to experiencing a serious adverse event that was attributable to the study intervention or procedure. These participants will be contacted via email within 72 hours of withdrawing from the trial with a list of crisis contact numbers for support. In this email, participants will also be asked to respond to the email (by clicking ‘reply’) if they would like to receive a phone call from the team clinical psychologist during business hours (9:00 to 17:00 AEST). If they say yes, they will be contacted within the following 72 hours.

Participants can self-withdraw at any time by submitting to the study team the UNSW standard withdrawal form appended to the Participant Information Statement. No mechanisms for automated withdrawal (e.g. via the study app) will be provided. To ensure that data belonging to a participant who withdraws can be uniquely identified, their mobile number and email address will be solicited on the withdrawal of participation form. Excluding self-withdrawals, the decision to withdraw a participant will be made by the Principal Investigator.

Handling of participant data after withdrawal will depend on when a participant decided to withdraw from the study. Participants will have their data removed from the study if they withdraw or are withdrawn prior to completion of the baseline assessment. Reflecting the intention-to-treat principles informing the planned analyses of the trial, data from participants who withdraw after this point will not be discarded.

Those who complete baseline and are randomly allocated but then complete no subsequent surveys will not be treated as withdrawal but handled as missing data unless the specifically request to have their data withdrawn. Responsibility for confirming successful withdrawal of data will be assigned in the Delegations Log.

Non-identifiable data about withdrawals enabling aggregate reporting (e.g. counts by intervention assignment) will be retained in the Withdrawals Log which is part of the Safety Monitoring Register (see Appendix B). In addition, any participant withdrawal linked to an adverse event will be recorded to enable reporting of safety-related statistics. Participants who withdraw from the study will not be replaced and will not be followed up except per adverse event follow-up, if applicable (see Risk Management Protocol, Appendix G).

# **Treatment** **of Subjects**

Participants will have access to the app for 120 days following completion of the baseline survey and can complete the modules in a self-guided way that best suits them. Users will be directed to each module linearly, unlocking a new module will require completion of the previous one. Once modules are unlocked, participants can re-access them at any time. Participants in the trial can use the app as often or as little as desired. App usage data will be collected directly through the LifeBuoy app, which will include the number of modules attempted and completed.

**Trial Intervention**


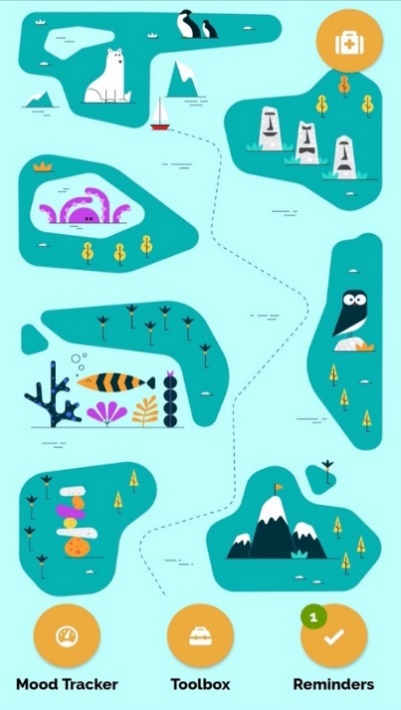
The intervention being trialled in this study is called *LifeBuoy*. *LifeBuoy* is a mobile treatment app developed by researchers at the Black Dog Institute. It is a fully automated, self-guided program theoretically grounded in Dialectical Behaviour Therapy (DBT) and Acceptance and Commitment Therapy (ACT). The app was initially developed using co-design approach, with young people with a lived experience of suicide, via online surveys and focus groups. The first version of LifeBuoy was evaluated in a trial which commenced in March 2020, with preliminary findings that it did lead to greater reductions in the severity of suicidal ideation in the intervention group, relative to the control group, at post-intervention – and these gains were maintained at 3-month follow up. The version of Lifebuoy being tested in this current trial has undergone further refinements based on qualitative feedback from trial participants, and in partnership with a youth Lived Experience advisory panel, recruited specifically for this purpose. The app contains seven learning modules that are based on Dialectical Behaviour Therapy (DBT), Acceptance and Commitment Therapy (ACT), and CT and incorporates wise mind principles (distress tolerance, emotion regulation/mindfulness, interpersonal effectiveness) through interactive learning exercises to help young people develop strategies and problem-solving skills for managing suicide thoughts.

The app also includes a ‘check-in’ feature that auto-appears when a user accesses the app for the first time each day or is accessible through the home screen. The check-in asks users how they are feeling today and if they select a negative mood, they are directed toward their safety plan, crisis contact numbers, and calming/distracting activities.

Participants will download the LifeBuoy app from the App Store or Google Play onto their personal smartphones. Once the app is downloaded, it will not require internet connection; internet connectivity will only be required to upload usage and adherence data to UNSW servers.

Participants will have access to the app - to complete seven modules - until the end of the 120 day trial period. Each module will take approximately between five to ten minutes to complete. The user will be directed to each module linearly and unlocking a new module will require completion of the previous one (this also is the gamification of the app, as island ‘light up’ or become technicolour when complete). Once modules are unlocked, participants can re-access them at any time.

In addition to the LifeBuoy app, this study is seeking to trial a multicomponent digital engagement strategy aimed at increasing compliance with the LifeBuoy app. The engagement strategy will be comprised of 3 core components:

1. An Instagram account that will deliver brief, visual content that aligns to the strategies and educational information within the app itself.
2. A clinician-written blog that will provide a platform to deliver in-depth therapeutic information and advice for maintaining mental health.
3. Emails: weekly emails will be sent to participants with links to strategies 1 and 2, to remind them to visit them and in addition, the emails will suggest to participants what module within the app they might like to visit/explore that week and strategies for getting the most out of it.

The decision to leverage these specific platforms was derived from a bottom-up approach, where we have spent almost 18 months gathering information and perspectives from young people with a lived experience of suicide. The sources of data include: qualitative interview feedback from participants in the first LifeBuoy trial (HC190764); (ii) data from a broad online survey of 260 mental health app users in Australia (HC200616) (iii) and input from a youth lived experience project advisory group (HC210038). The content and design for the Instagram and blog posts is being developed in consultation with the youth lived experience project advisory group, all of whom were participants in the first LifeBuoy trial.

To deliver on the engagement strategy, Instagram posts will be uploaded at least once a week throughout the duration of the study, up till 30 days after recruitment of the final study participant. The Instagram account will be public, with comments disabled, as public access allows users to view content anonymously (otherwise they will need to register to view the account, which may be perceived as a barrier to engagement).

For the blog, new articles will be posted once a week throughout the duration of the study, again, up to 30 days following the recruitment of the final participant.

# **Types of Safety and Monitoring**

Assessment of Safety Event Report Forms

Safety reports will be assessed on the seriousness, causality, and expectedness of the event to the trial treatment(s), intervention(s), investigational medical product(s), investigational medical device(s). The following are known and expected adverse effects, harms, risks or discomforts associated with trial procedures, treatments or interventions.

Known Adverse Effects

An individual scoring above 20 on the SIDAS at any timepoint will be considered an expected adverse event (given the study population has a history of recent suicidal ideation).

Known Harms, Risks or Discomforts

Anxiety induced by answering a questionnaire and/or engaging in therapeutic activities on the app, and the cessation of updates from the digital engagement strategies at the end of the data collection phase. There is a moderate likelihood and low severity of this discomfort occurring, given the more than low-risk population participating in the study.

Psychological harms, including feelings of distress or anxiety, for example, due to disclosure of sensitive information on questionnaires and/or engaging in therapeutic activities on the app. There is a moderate likelihood and low severity of this discomfort occurring, given the more than low-risk population participating in the study.

Adverse Events or Adverse Reactions: general definitions

Adverse events (AE) are considered any untoward medical occurrence in a clinical trial participant administered the intervention, which does not necessarily have a causal relationship with this treatment.

Adverse Reactions (AR) are considered untoward and unintended responses that is related to the trial intervention or intervention procedures.

AEs and ARs are assessed using the safety monitoring flow chart. Those classified as "not serious" are assessed by the qualified physician/medical expert specified in section 2 of the protocol. The Qualified Physician cannot delegate this responsibility to other research personnel.

Adverse event reports must be reported to the Coordinating Principal Investigator within 24-hours. All adverse event reports must be recorded in the [UNSW Safety Monitoring Register Template](https://research.unsw.edu.au/document/UNSW%20Safety%20Monitoring%20Register%20Template.xlsx).

Serious Adverse Events

In this trial a Serious Adverse Event (SAE) is o one or more of the following events and the event is not related to the trial intervention:

1. A participant reporting a suicide attempt or NSSI that requires medical care at any follow-up assessment time point.
2. If we become aware of a death of a participant during the trial, for any reason, this will also be considered a serious adverse event.

#### **Serious Adverse Reaction**

To assess whether the serious adverse event was related to the trial or intervention, participants who report a recent suicide attempt or NSSI that required medical care at any follow-up assessment time points will be asked a follow-up question: “Do you think that participating in this study or using this app has contributed to any recent distress leading you to self-harm?”

A serious adverse reaction by its nature, incidence, severity, or outcome is anticipated and identified in the current version of the intervention safety information are classified as a SAR report. SAR reports are reported to the Coordinating Principal Investigator within 24-hours for multicentre clinical trials. Serious Adverse Reaction reports must be recorded in the [UNSW Safety Monitoring Register Template](https://research.unsw.edu.au/document/UNSW%20Safety%20Monitoring%20Register%20Template.xlsx).

#### **Suspected Unexpected Serious Adverse Reaction (SUSAR)**

A serious adverse reaction by its nature, incidence, severity, or outcome is unanticipated and not identified in the intervention’s instructions for use or safety information are classified as a SUSAR.

Fatal or life-threatening Australian SUSAR reports are reported to the Coordinating Principal Investigator, the sponsor's delegate and the approving HREC within 7 calendar days after being made aware of the case follow up information reported within a further 8 calendar days.

All other Australian SUSAR reports are to be reported to the Coordinating Principal Investigator, the sponsor's delegate and the approving HREC within 15 calendar days after being made aware of the case follow up information reported within a further 8 calendar days. SUSAR reports must be recorded in the [UNSW Safety Monitoring Register Template](https://research.unsw.edu.au/document/UNSW%20Safety%20Monitoring%20Register%20Template.xlsx).

Participants may be withdrawn from the study if they experience a serious adverse event that is attributable to a study intervention or procedure; or means they can no longer participate in the study. The team clinical psychologist will follow-up with subjects withdrawn due to experiencing a serious adverse event that was attributable to the study intervention or procedure. These participants will be contacted via email 1-week after they are withdrawn from the trial to provide a list of crisis contact numbers for support. In this email, participants will also be asked to respond to the email (by clicking ‘reply’) if they would like to receive a phone call from the team clinical psychologist during business hours (9:00 to 17:00 AEST) within the next 3 days. SAE reports are classified following the safety assessment flowchart and are assessed by Sponsors Independent Medical specified in section 2 of the protocol. The Sponsors Independent Medical cannot delegate this responsibility to other research personnel. SAE reports are reported to the Coordinating Principal Investigator within 24-hours for multicentre clinical trials. SAR reports must be recorded in the [UNSW Safety Monitoring Register Template](https://research.unsw.edu.au/document/UNSW%20Safety%20Monitoring%20Register%20Template.xlsx).

Significant Safety Issue (SSI)

A safety issue that could adversely affect participants' safety or materially impact the trial's continued ethical acceptability or conduct. The Human Research Ethics Committee and Sponsor's Delegate must be notified of all significant safety issues within 15 calendar days of the sponsor instigating or being made aware of the issue**.** SSI reports must be recorded in the [UNSW Safety Monitoring Register Template](https://research.unsw.edu.au/document/UNSW%20Safety%20Monitoring%20Register%20Template.xlsx).

Register of Clinical Trial Safety Monitoring Reports

A register of all event reports assessed and classified is to be retained by the Coordinating Principal Investigator and reported to the trial sponsor annually and the HREC if required.

Reporting of Clinical Trial Safety Monitoring Reports

Single case reports of Adverse Events Adverse Reactions, Serious Adverse Events (SAEs), Serious Adverse Reactions (SARs), reports do not need to be reported to the UNSW Sponsor's Delegate or the HREC. All single case reports must be recorded in a safety monitoring register and are reported to the UNSW Sponsor's Delegate annually.

# **Non-compliance, Protocol Deviation and Serious Breaches of Good Clinical Practice**

## **Protocol Deviation**

A protocol deviation is defined as any breach, divergence or departure from the requirements of Good Clinical Practice, the clinical trial protocol, the clinical trial standard operating procedures, or the human ethics approval that does not have a significant impact on the continued safety or rights of participants or the reliability and robustness of the data generated in the research or clinical trial. Protocol deviations are events that do not occur persistently or systematically and do not potentially result in participant harms. Examples of protocol deviations include but are not limited to:

- Deviations because of participant adherence to the protocol, including rescheduled study visits, participants refusal to complete scheduled research activities
- The completion of consent forms, safety monitoring report, case report forms or data collection tools in a manner that is not consistent with the protocol instructions or failure to make reports within the required reporting timeframes.
- Use of an unapproved version of the participant information statement or recruitment of participants using unapproved recruitment procedures.
- Inclusion of a participant that does not meet the inclusion criteria.
- An urgent safety measure must be taken to eliminate an immediate hazard to a participant's health or safety.

##

## **Serious Breach of Good Clinical Practice**

A serious breach is defined as a breach of Good Clinical Practice, the clinical trial protocol, the clinical trial standard operating procedures, or the human ethics approval that is likely to affect to a significant degree the safety or rights of participants or the reliability and robustness of the data generated in the clinical trial. Examples of serious breaches include but are not limited to:

- Persistent or systematic non-compliance with the instructions for completing consent forms, safety monitoring forms, case report forms or data collection tools that result in continued missed or incomplete data collection.
- Failure to record or report adverse events, serious adverse events, suspected unexpected serious adverse reactions, significant safety issues where urgent safety measures were implemented.
- Failure to conduct clinical trial procedures following the clinical trial delegation log.
- Widespread and uncontrolled use of protocol waivers affecting eligibility criteria, which leads to harm to trial subjects.
- Failure to report investigational medical product or device defects to the clinical trial sponsor or any relevant regulatory body.
- Failure to conduct research following the issued approvals, permits or licences by required laws, regulations, disciplinary standards, and UNSW policies relating to the responsible or safe conduct of research.
- Concealing or facilitating breaches (or potential breaches) of the Research Code by others.
- Researching without the requisite approvals, permits or licences required by laws, regulations, disciplinary standards, and UNSW policies related to the responsible or safe conduct of research.
- Failure to conduct research as approved by an ethics review body where that conduct leads to (or has the potential to) results in participant harms.
- Researching without ethics approval as required by the National Statement on Ethical Conduct in Human Research where that conduct leads to (or has the potential to) result in participant harms.
- Any breaches as outlined in the UNSW Research Misconduct Procedure or the Australian Code for responsible conduct of research that leads to (or can potentially) result in participant harms.

## **Reporting Protocol Deviations**

- Protocol deviations occurring at a site must be documented in site files and reported by the principal site investigator to the Coordinating Principal Investigator.
- The Coordinating Principal Investigator must review the protocol deviation and the clinical trial protocol to establish the corrective actions and preventative steps to prevent the deviation from reoccurring.
- The protocol deviation and corrective action plan must be reported to the UNSW Sponsor's Delegate by the Coordinating Principal Investigator or Coordinating Research Team using the protocol deviation report form.

## **Reporting of a Serious Breach**

- The Principal Investigator must report a serious breach occurring at a participating site to the Coordinating Principal Investigator within a specified timeframe.
- The Coordinating Principal Investigator must review the serious breach, along with the clinical trial protocol, to develop a Corrective and Preventive Action (CAPA, Appendix L) that defines the steps to prevent the serious breach from reoccurring.
- The serious breach report and the CAPA must be provided to the approving HREC, and the UNSW sponsors delegate for review and approval.

# **Review of a Protocol Deviation and a Serious Breach**

- The UNSW Sponsor's Delegate will review reports to establish whether the event meets the definition of a protocol deviation or serious breach, establish whether the proposed CAPA is appropriate and establish whether there is or will be ongoing impact reliability and robustness of the data generated.
- The UNSW Sponsor's Delegate will seek advice from the approving HREC on the corrective and preventive actions.
- Protocol deviation or serious breach reports where a UNSW researcher, staff or student is responsible for the protocol deviation or the serious breach will be reviewed as per the [UNSW Research Misconduct Procedure](https://www.gs.unsw.edu.au/policy/documents/researchmisconductproc.pdf) to establish a breach of the [UNSW Research Code of Conduct](https://www.gs.unsw.edu.au/policy/documents/researchcode.pdf) has occurred.
- Protocol deviation or serious breach reports where the UNSW Sponsor's Delegate determines that site personnel are responsible for a protocol deviation or the serious breach will be referred onto their responsible institution for review under their Research Misconduct procedures to establish whether a breach of the [Australian Research Code for the Responsible Conduct of Research](https://www.nhmrc.gov.au/about-us/publications/australian-code-responsible-conduct-research-2018) has occurred.

# **Statistical analytical plan**

Mixed models repeated measures (MMRM) analyses, with maximum likelihood estimation and an appropriate covariance structure, will be used to evaluate longitudinal changes in suicidal ideation and secondary mental health outcomes between the LifeBuoy and attentional control arms. Where required, generalised linear mixed models with an appropriate link function will be used for the analysis of categorial variables. The primary outcome is severity of suicidal ideation, as measured by the Suicidal Ideation Attributes Scale [SIDAS], over time (baseline T0, 30-days T1, 60-days T2, and 120-days T3). The mixed model approach incorporates all available data, including participants with missing follow-up data points, under the missing-at-random assumption. Analyses will therefore accord with the intention-to-treat principle.

Descriptive statistics will be used to examine the level of engagement in three arms (LifeBuoy, LifeBuoy + engagement, and control). Engagement will be measured by the number of modules completed by participants. Participant module access and module completion will be automatically collected via the app.

Qualitative feedback on users’ perceived acceptability of the LifeBuoy app and engagement strategy will be examined through semi-structured interviews with a subset of participants (n=30 in total) and questionnaires in the survey. The interview data will be analysed using thematic analysis. An inductive approach, independent of a theoretical confirmative method will be used to identify and group themes. The researchers will then refine the themes and determine the final coding framework. Discrepancies will be resolved by a third researcher to ensure reliability of the process.

Any deviation from the original statistical plan will be described and justified in the final report.

# **Data Ownership**

All research data collected during this trial is governed and handled following the Research Data Governance and Materials Handling [policy](https://www.gs.unsw.edu.au/policy/documents/researchdatagovernancepolicy.pdf). UNSW, rather than any individual or Organisational Unit, is the Custodian of data and materials and any information derived from the data. Original research data and primary materials generated in the research conducted at the University will be owned and retained by the University subject to any contractual, statutory, ethical, or funding body requirements.

# **Handling and Reporting Data**

Principal Investigators are responsible for maintaining adequate and accurate source documents and trial records that include all pertinent observations on each site's trial subjects. Source data must be attributable, legible, contemporaneous, original, accurate, and complete.

Trial subjects will be assigned a participant ID, and data will be reported using the case report form (see Appendix B). Data reported on the case report form, derived from source documents, should be consistent with the source documents, or the discrepancies must be explained. Any change or correction to a case report form should be dated, initialled, and explained (if necessary) and should not obscure the original entry (i.e., an audit trail should be maintained); this applies to both written and electronic changes or corrections.

Direct Access to Source Data and Documents

Principal investigator(s) and institution(s) will permit trial-related monitoring, audits, IRB/IEC review, and regulatory inspection(s), providing direct access to source data/documents.

Monitoring Quality Control and Quality Assurance

The Coordinating Principal Investigator and Principal Investigator(s) 'responsibility are to monitor the clinical trial. The Coordinating Principal Investigator and Principal Investigator(s) are responsible for undertaking or participating in site initiation or protocol-specific training before recruitment and data collection commences. A monitoring report demonstrating regular compliance monitoring with the clinical trial protocol, procedures, and HREC approval is provided to the UNSW Sponsor's Delegate annually.

Root, cause, analysis reports are to be completed by the Coordinating Principal Investigator for reports of non-compliance and serious breaches. A corrective and preventative action plan must be developed and actioned for any reports of non-compliance and serious breaches.

# **Essential Documents for the Conduct of a Clinical Trial**

All essential documents referred to in section 8.2 of the [Guideline for Good Clinical Practice (E6, R2)](https://database.ich.org/sites/default/files/E6_R2_Addendum.pdf)   are to be retained by all trial investigators.

# **Appendix**

# Clinical Trial Delegation and Responsibilities Log

| **Protocol / Study Number:** | HC210400 | **Sponsor Name:** | Ted Rohr |
| --- | --- | --- | --- |
| **Principal Investigator Name:** | Dr Michelle Tye | **Site Number:** | N/A |
| **Site Name (if applicable)** | N/A | | |
| **Version (date)** | 1.0 (6 July 2021) | | |

***THIS FORM IS TO BE COMPLETED BY ALL PERSONNEL INVOLVED IN THE STUDY AFTER RECEIVING PROPER STUDY TRAINING AND BEFORE TAKING PART IN ANY STUDY ACTIVITIES**

**Principal Investigator (PI)**

By signing, I confirm/acknowledge that the tasks listed below will only be delegated to appropriately trained, skilled and qualified staff. I will remain responsible for the overall study conduct and reported data, ensuring study oversight. All associates, colleagues, and employees assisting in the conduct of the study are informed about their obligations and have not performed any study tasks before appropriate delegation and completion of appropriate training. Mechanisms are in place to ensure that site staff receives the appropriate information and training throughout the study and that a 2-way communication channel exists between staff and self. Any changes in staff or delegation in staff will be recorded promptly.

| **Name** | **Principal Investigator’s Signature** | **Initials** | **Start**  **(dd/mmm/yyyy)** | **End**  **(dd/mmm/yyyy)**  **(complete only if prior to end of study)** |
| --- | --- | --- | --- | --- |
| Dr Michelle Tye |  |  |  |  |

Site Staff

| **Name** | **Signature** | **Initials** | **Study Role** | **Key Study Task(s)** | **Start**  **(dd/mmm/yyyy)** | **End**  **(dd/mmm/yyyy) (complete only if prior to end of study)** | **PI Initials & Date**  **(dd/mmm/yyyy)** |
| --- | --- | --- | --- | --- | --- | --- | --- |
| Dr Michelle Tye |  | MT | Principal Investigator | Overall responsibility for the conduct, oversight and monitoring for the trial. | 01/SEP/2021 |  | __/___/_____ |
| Dr Lauren McGillivray |  | LM | Co-chief investigator and Project Manager | Administration of the Delegations Log, oversight of day-to-day trial management, participant recruitment, completion and documentation of pre-trial and trial-initiation monitoring procedure, active monitoring of study email address for participant risk disclosures (must be delegated during absence), ensuring timely follow-up of all participant risk disclosures as per the Psychological Safety Response Procedure, maintenance of Safety Monitoring Register and ensuring timely completion of Unexpected and Serious Adverse Event and Significant Safety Issue notification forms. | 01/SEP/2021 |  | __/___/_____ |
| Dr Quincy Wong |  | QW | Investigator | Provide expertise in trial design and execution, data analysis, and interpretation of data. | 01/SEP/2021 |  | __/___/_____ |
| Dr Jin Han |  | JH | Investigator | Provide expertise in trial design and execution, data analysis, and interpretation of data, provide expertise relating to the use and delivery of the LifeBuoy app, maintenance of secured randomisation list, codebreaking on study completion or if required by the Data Safety Monitoring Board. | 01/SEP/2021 |  | __/___/_____ |
| Mr Daniel Gan |  | DG | Student Investigator | Assisting the Project Manager with Day-to-day trial operations, verification and analysis of screening and trial data. | 01/SEP/2021 |  | __/___/_____ |
| Associate Professor Sarah Hetrick |  | HC | Investigator | Provide expertise in running of the trial, interpretation of data, and clinical guidance of safety procedures. | 01/SEP/2021 |  | __/___/_____ |

*These tasks may only be performed by qualified individual as permitted by local law, medical or standard of care practices, or applicable required training as per job description or designation.

# **Data and Safety Monitoring Board Charter**

A randomised controlled trial of an m-health app and digital engagement strategy for improving treatment adherence and reducing suicidal ideation.

| Sponsor: | University of New South Wales, Sydney |
| --- | --- |
| HREC Reference number: | HC210400 |
| BDI Study Identification Number: | 247 |
| Clinical Trials Registration Number: | ACTRN12621001247864 |
| Number of Sites: | One (Australia) |
| Number of Participants: | 669 |

**Data and Safety Monitoring Board (DSMB) Overview**

**DSMB Description**

This DSMB members will have a degree of independence from the Black Dog Institute, University of New South Wales Sydney, and investigators, as required by the NHMRC for non-commercial trials.

This charter will be approved by its DSMB members.

The DSMB will function in accordance with the principles of the following documents: Good Clinical Practice (GCP) Guidelines, Declaration of Helsinki 2000, NHMRC National Statement on Ethical Conduct in Human Research, NHMRC Guidance Safety and Monitoring of Clinical Trials involving a Therapeutic Good, and University of New South Wales HREC guidelines.

**DSMB Membership Conflicts of Interest & Composition**

Members will disclose conflicts of interest and will be cleared of significant conflicts of interest and potential conflicts of interest. No member should have financial, proprietary, professional, or other interests that may affect impartial, independent decision-making by the DSMB.

Composition of membership will reflect expertise in clinical trials (including a good understanding of the problems and limitations of trials), statistics and the specific scientific expertise relevant to the study, in this case, the suicidality in young people. Members will comprise 3 individuals with expertise in these areas.

A quorum group of two must be present for closed sessions and subsequent decisions and/or recommendations made by the committee.

**Reporting**

Data requiring review by the DSMB will be provided by the Trial Manager (Dr Lauren McGillivray) for the open session, and the data officer (TBC) for the closed session.

Issues and recommendations identified by the DSMB will be provided to Trial Steering Group (TSG, Appendix L), comprising the Trial Lead and Chief Investigator (Dr Michelle Tye), the Trial co-lead and Investigator/Trial Manager (Dr Lauren McGillivray), and the co-Investigators (Dr Quincy Wong, Prof Helen Christensen, Dr Jin Han, and Mr Daniel Gan) by the DSMB in accordance with this charter.

**Study Summary**

Involving young people aged 17 – 24 years from the general community in Australia, this randomised controlled trial (RCT) will examine the use of a smartphone application – LifeBuoy - to reduce symptom severity of suicidal ideation. Concurrently, this study also examines whether a digital engagement strategy promotes greater use of the Lifebuoy app. This study will rely on self-report data to evaluate the efficacy of the app, and on automated system data to evaluate the efficacy of the digital engagement strategies. This study is funded by Australian Rotary Health, the Roth Family Foundation, the Mantana Foundation for Young People, and the Goodman Foundation.

**Design**

A double-blind, randomised-controlled trial with three-parallel arms (an intervention arm, intervention + engagement strategy arm, and an attentional control arm) with 1:1:1 allocation. Assessment and study procedures are automated and so the risk of differential treatment of those in the intervention and control is minimised.

**Objectives**

Comparing 2-arms, we will assess the efficacy of the LifeBuoy app in reducing suicide ideation among young people, as measured by the Suicidal Ideation Attributes Scale (SIDAS; [9]), relative to a placebo attention control condition. Changes in suicidal ideation will be assessed by comparing the intervention to the attention placebo control condition at 0-days (baseline, T0), 30-days (T1), 60-days (T2), and 120-days (T3). In addition, the trial will be used to:

- Examine the efficacy of a digital engagement on app adherence (measured by the number of modules / activities / exercises completed; number and frequency of app log-ins; total time spent on the app) by comparing 2-arms: LifeBuoy + engagement strategy versus LifeBuoy-only at T1, T2, and T3.
- To determine whether the LifeBuoy app reduces incidents of suicide attempt and non-suicidal self-injury relative to the placebo attention control condition at T1, T2, and T3.
- To assess whether the LifeBuoy app reduces depression and anxiety symptoms relative to the placebo attention control condition at T1, T2, and T3.
- To examine whether the LifeBuoy app + engagement condition reduces suicidal ideation relative to the LifeBuoy-only condition at T1, T2, and T3.

**Investigational Products**

LifeBuoy. The Lifebuoy app is a mobile app developed by researchers at the Black Dog Institute. It is a fully automated, self-guided program for young people experiencing mild-to- moderate symptoms of suicidal ideation. The app has been developed using a person-centered approach, to understand and accommodate the perspectives of young people who will use the intervention. Participants will download the app from the App Store or Google Play onto their personal smartphones. Once the app is downloaded, it will not require internet connection; internet connectivity will only be required to upload usage and adherence data to UNSW servers. The app contains seven learning modules derived from third wave CBT, and incorporates wise mind principles (distress tolerance, emotion regulation/mindfulness, interpersonal effectiveness) through interactive learning exercises to help young people develop strategies and skills for managing emotions and distress. Participants have access to the app until the end of the trial (120 days). Each module is expected to take 5 to 10 minutes to work through. The user will be directed to each module linearly.

**Follow-up Schedule**

All participants will be asked to complete self-report questionnaires at T1 – T3.

**Roles and Responsibilities**

As outlined by NHMRC Guidance on “Safety monitoring and reporting in clinical trials involving therapeutic goods” (2016), the roles of the DSMB will be to assist in:

- safeguarding the interests of study participants
- ensuring that definitive and valid results are produced which will reliably inform future healthcare decisions enhancing the credibility of the trial.
- These roles will be achieved by examining the data accumulated during the progress of the trial, ensuring risks and benefits are monitored, and that the trial remains safe for participants. The role of the committee is to review data completeness, adverse events, outcome data (unblinded if there is good reason for this) and to recommend to the trial committee whether the study requires modification.
- Recommendations will be made to the TSG, which, if appropriate, will be shared by the trial team with the Sponsor and the HREC.

Following each assessment point, the DSMB will:

- Review aggregate subject data related to safety, attrition, withdrawals, data integrity and overall conduct of the trial and discuss via telephone conference.
- Provide recommendations to continue, modify or terminate the trial.
- Maintain records of all activities.
- Comply with conflict of interest guidelines and confidentiality guidelines as described in this charter.

The Investigators will:

- Assure the proper conduct of the study according to the study protocol and relevant research guidelines;
- Assure collection of accurate and timely data;
- Compile and report data for the DSMB report at the end of each assessment period (trial manager and data officer);
- Monitor the project email inbox;
- Promptly report potential safety concern(s) to the DSMB;
- Communicate with regulatory authorities, e.g. HREC, in a manner that maintains integrity (e.g., blinding) of the data, as necessary.

**DSMB Membership**

**Composition**

DSMB membership for the trial will be established prior to recruitment commencing and an updated charter listing the DSMB members will be submitted to the Ethics Review Committee.

**Selection of the Chair**

The Chair should have previous experience of serving on a DSMB and experience of chairing meetings, and should be able to facilitate and summarise the DSMB discussions.

**DSMB Meetings**

**The first meeting**

Prior to recruitment commencing, the DSMB members will review and ratify this charter and form an understanding of the protocol and study endpoints.

The members should review this charter and form an understanding of the protocol and study endpoints. Meetings will take place at an appropriate regularity decided by the DSMB committee. The DSMB will also meet if evidence from other research groups suggests potential for risk.

**Meeting formats**

DSMB meetings will be booked in for the week following the completion of data collection at each assessment point of the trial (baseline, 30-days, 60-days, and 120-days). Procedurally, when these set meetings are booked they will include both an open and closed session, however, it is up to the DSMB to decide if they would like to have a closed session. The typical structure of the meetings will first start with an open session involving both DSMB members, the trial leads, and the trial manager, followed by the closed session if needed. The meetings will be organised and chaired by either MT or LM. These meetings will generally be conducted by teleconference or videoconference, but the DSMB can decide whether they would like to do this face-to-face.

In the event of a closed meeting, unblinded data may be requested if there is a rationale (e.g. unexpected adverse events, aggregate data indicates self-harm above the rates expected from this group). Only DSMB members and others whom they specifically invite are present in closed meetings.

Outside of the pre-scheduled meetings, an extraordinary meeting may be called if a serious adverse event occurs. The trial leads or trial manager will report the event to the Chair within 24 hours of becoming aware of the event, and the Chair will convene a closed session meeting to discuss the event and make recommendations.

Following each meeting, a report minuting the open session including any DSMB recommendations and rationale for such will be prepared by the minute taker and sent to the CIA (MT) and all other members. For closed sessions, recommendations will be prepared by the Chair and sent to the Trial Lead (LM), who will present the outcomes at the TSG following each meeting. Meetings will occur until the trial concludes.

**Reporting**

As this is a trial of an mhealth preventive intervention, and not a medicine or treatment, it is regarded as being in the lowest risk category, type A, with risk comparable to standard medical care. All Serious Adverse Events (SAEs) will be regarded as non-expedited (ie. only to be reported at the next DSMB meeting), unless the SAE is both unexpected and possibly related directly to participation in the trial, in which case it will be reported to the DSMB via email within 24 hours as well as other agencies. For this 6-week low intensity app-based DBT intervention, it is anticipated that all SAEs that are possibly related to trial participation will be reported. In the event of uncertainty, the TSG should default to reporting the SAE to the DSMB.

**Responsibilities of the Data Officer**

The data officer will be responsible for producing the closed session report to the DSMB, and assist the trial manager with the open session report. The main purpose of this role is to ensure that the Investigator team remain blind to study outcome data. No interim analyses will be performed unless explicitly required by the DSMB with a strong rational. The process for communicating the report is as follow:

1. The data officer will extract unblinded data and prepare reports for the DSMB (using the attached shell) and disseminate the report one week prior to the scheduled meeting.
2. The data officer will be responsible for extracting both confidential and non-confidential data from the trial data collection platform.
3. The data officer will participate in the DSMB meetings to take the members through the report if required.
4. These discussions will remain confidential and not communicated to the trial team.
5. Responsibilities of the Chief Investigator and Trial Steering Group (TSG)
6. The trial leads and trial manager must be available to attend open sessions.
7. Other TSG members (see membership details on p. 5) are not required to attend, but may attend if they wish to.

**Safety Analyses**

Suicidal behaviour (ideation, attempts, and NSSI)

- Suicidal ideation, as measured by % scoring >20 on the SIDAS
- A suicide attempt during the trial identified at all follow up surveys: “Since the last LifeBuoy study survey, how many times have you made an attempt to kill yourself in which you had some intent to die?” >0 (T1 – T3) or ad hoc self-report by participants to research study email.
- Non-suicidal self-injury during the trial identified at all follow up surveys: “Since the last LifeBuoy study survey, how many times have you injured yourself on purpose without suicidal intent (i.e., intentional, self-inflicted damage to the surface of the body but with no intent to die)?” >0 (T1 – T3) or ad hoc self-report by participants to research study email.
- Serious adverse events (including all events requiring medical intervention).
- Enrolment data, intervention completion data, attrition data, adverse event data, and other administrative data.

The TSG cannot access the unblinded data for suicidal ideation, attempt, or NSSI as these are outcome measures and will be shared only in a closed session.

**Trial Stopping Guidelines**

The DSMB has the responsibility for deciding whether the trial should be stopped at any stage. They will do this if, and only if, two conditions are satisfied:

1. The results provide proof beyond reasonable doubt that the intervention is on balance definitely harmful, or for a particular category of, participants in terms of the major outcome; or is on balance, definitely having a therapeutic effect (noting that this is extremely difficult to assess without long-term follow-up periods).
2. The TSG are not conducting the project safely, which would include, but is not limited to, a failure to contact participants who flag as ‘at risk’ (scores >20 on the SIDAS, suicide attempt during the trial, or NSSI requiring medical care during the trial) within the 48 hour period specified in the Duty of Care section within the Trial Protocol.

There is no plan to conduct any interim analyses, unless in cases where there is justification to unblind the data and condition (1) above is met. If this occurs, this DSMB Charter aligns with the Peto-Haybittle stopping rule whereby an interim analysis of a major endpoint would generally need to involve a difference between treatment and control of at least three standard errors to justify premature disclosure. An interim subgroup analysis would have to be even more extreme to justify disclosure. This rule has the advantage that the exact number and timing of interim analyses need not be pre-specified. In summary, the stopping rules require extreme differences to justify premature disclosure and involve an appropriate combination of mathematical stopping rules and scientific judgment.

**DSMB Documentation and Communication**

Available information at open sessions. This will include routine study information (consent, completion, attrition), risk alerts, follow-up data, by group.

**Available information at closed sessions**

Unblinded data for SIDAS and suicide attempt and NSSI data (including effect size estimates).

**Blinding**

If necessary, the DSMB will be provided unblinded data by the data officer.

**Access to unblinded data**

Only the DSMB members, trial leads and the trial manager have access to this data. The DSMB cannot share this beyond the committee to the trial team.

Who is responsible for circulating external evidence (e.g., from other trials, population-based prevalence estimates)? Trial manager, Lauren McGillivray.

**To whom will the DSMB communicate decisions and recommendations?**

Within two weeks following each meeting, the DSMB will send findings and recommendations to the Trial Steering Group (TSG).

**How are responses communicated back to the DSMB?**

The TSG (Co-chaired by MT or LM) will review and respond to the DSMB recommendations if required. If the recommendations request action, the PI will provide a written response stating whether the recommendations will be followed and the plan for addressing the issues. Upon receipt, the DSMB will consider the TSG response and will attempt to resolve relevant issues, resulting in a final decision. The investigator will agree to disseminate the final decision to the appropriate regulatory agencies (TGA and HREC) within an appropriate time.

**What happens to the reports after each DSMB meeting?**

All DSMB members are asked to destroy reports after each meeting. Internal records will be kept by the data manager. Each report will add a table onto the previous one, and as such the reports will be cumulative.

**What happens to minutes and records?**

Any minutes should be kept by the Chairs until after the final trial analysis is complete, upon which point they should be shared with the Trial Manager for archiving for a minimum of 7 years.

| Participant Information Sheet, Consent Form, Withdrawal form.  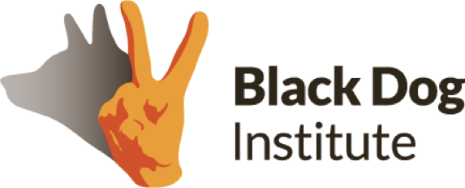 | 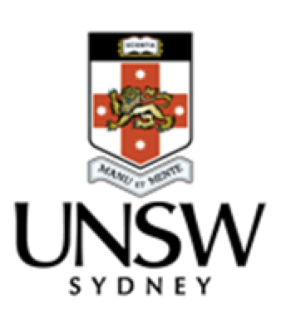 |
| --- | --- |
| **PARTICIPANT INFORMATION STATEMENT AND CONSENT FORM** | |
| **A randomised controlled trial of an m-health app and digital engagement strategy for**  **improving treatment adherence and reducing suicidal ideation.**    **Chief Investigator: Dr Michelle Tye** | |

1. **What is the research study about?**

You are invited to take part in this research study. The research study aims to find out if a mental health app - LifeBuoy - is effective in helping young people manage their suicidal thoughts. We also wish to find out if a digital engagement strategy helps to promote use of the app.

1. **Who is conducting this research?**

The study is being carried out by the following researchers: Dr Michelle Tye (Principal Chief Investigator), Dr Lauren McGillivray (Chief Investigator and Project Manager) and Mr Daniel ZQ Gan (Student Investigator) from the Black Dog Institute, University of New South Wales.

**Research Funder:** This research is being funded by Australian Rotary Health, The Roth Family Foundation, Mantana Foundation for Young People, and The Goodman Foundation. None of the funding organisations are involved in this research study, nor do they have access to the information obtained through this study.

1. **Inclusion/Exclusion Criteria**

Before you decide to participate in this research study, we need to ensure that it is ok for you to take part. The research study is looking recruit people who meet the following criteria:

1. 17 to 24 years of age
2. Living in Australia
3. Fluent in English
4. Own and have access to a smartphone
5. Have experienced suicidal thoughts in the past 30 days

Participants who meet the following criteria will be excluded from the study:

1. Have had a diagnosis of psychosis or bipolar disorder in the past 30 days (because of increased risk to this vulnerable group)
2. **Do I have to take part in this research study?**

Participation in this research study is voluntary. If you do not want to take part, you do not have to. If you decide to take part and later change your mind, you are free to withdraw from the study at any stage.

If you decide you want to take part in the research study, you will be asked to:

- Read the information carefully (ask questions if necessary);
- Sign and return the consent form if you decide to participate in the study;
- Take a copy of this form with you to keep.

1. **What does participation in this research require, and are there any risks involved?**

If you agree to participate you will be asked to complete the following research procedures.

**Screening:** Complete a 1-minute online screening survey, which will ask if you meet the study inclusion/exclusion criteria. If the screening survey shows that you meet the criteria for inclusion, then you will be able to participate in this research study. If the screening survey shows that you do not meet the criteria for inclusion, then you cannot participate and will be directed to a webpage that will include relevant support contacts.

**Randomisation:** The aim of the research is to examine the effects of two (app-based) digital interventions for the management of suicidal ideation, which may include elements of education, advice, and skills-based strategies, as well as mood tracking. A computer program will be used to randomly allocate you into a group.

**App Use:** You will be asked to download the app on your phone, and you will have access to the app until the end of the trial (in 120-days). Some user activity will be recorded by the app, including which modules you attempt and complete, and the time(s) of your log-ins. This activity will be accessible to the research team only.

**Questionnaires:** Online questionnaires asking you about your mental health will need to be completed on four occasions: baseline (day 0), 30-days, 60-days, and 120-days. Each questionnaire takes about 20 minutes to complete. The research team will contact you via email or SMS to remind you when to complete each questionnaire.

**Interview:** You may also be selected to participate in 30-45 minute interview during the study. Participant spots will be limited and scheduled in on a first-in, first-served basis. You will be asked questions about your experience with the intervention. Understanding your user experience is important for knowing why it may or may not have worked for you and help us to refine the intervention. The interview will take place via telephone or videoconferencing, depending on your preference. With your permission, the research team would like to audio record the interview. If you do not wish to be recorded but you would like to participate, please advise the research team and written notes will be taken. At the start of the interview, participants will be asked to reconfirm verbally that they consent to participate in the interview.

**Reminders:** You will be sent a maximum of two reminder notifications to complete the questionnaires at each time point via email and/or SMS.

**Additional Costs and Reimbursement:** Mobile data is not required to use the app, but it is required to initially download the app. You will receive a $10 e-gift card after completing surveys at day-30, day-60, and day-120, equalling up $30 in total. If you participate in an interview, you will be reimbursed a $30 e-gift card.

**Psychological Distress:** You may feel that some of the questions we ask are stressful or upsetting. If you do not wish to answer a question, you may skip it and go to the next question, or you may stop immediately. Regardless of which app you receive, we have included a “help button” that contains a directory of key sources of help such as phone numbers for Kids Helpline and LifeLine to ensure that you have access to support 24/7.

1. **What will happen to information about me?**

By signing the consent form, you consent to the research team collecting and using information about you for the research study. The research team will store the data collected from you for this research project for a minimum of 15 years after publication of the research. The information about you will be stored in a re-identifiable format where any identifiers such as your name, address, date of birth will be replaced with a unique code.

You will be asked to provide your consent for the research team the share or use the information collected from you in future research that will be an extension of, or is closely related to, the original project; or is in the same general area of research.

Your information will only be shared in a format that will not identify you.

- Information collected from you in an electronic format stored on a UNSW password protected OneDrive. This It is stored on UNSW servers (MED/BDI) protected by encryption, and only accessible to the approved research investigators.
- If applicable, audio recordings will be stored on a UNSW password protected OneDrive only accessible to the approved research investigators.

The information you provide is personal information for the purposes of the Privacy and Personal Information Protection Act 1998 (NSW). You have the right of access to personal information held about you by the University, the right to request correction and amendment of it, and the right to make a complaint about a breach of the Information Protection Principles as contained in the PPIP Act. Further information on how the University protects personal information is available in the [**UNSW Privacy Management Plan**](https://www.legal.unsw.edu.au/compliance/privacyhome.html).

1. **How and when will I find out what the results of the research study are?**

The research team intend to publish and/ report the results of the research. All Information will be published in a way that will not identify you. If you would like to receive a copy of the results you can let the research team know by inserting your email or mailing address in the consent form. We will only use these details to send you the results of the research.

1. **What if I want to withdraw from the research study?**

If you do consent to participate, you may withdraw at any time. You can do so by contacting the research team and tell them you no longer want to participate. Your decision not to participate or to withdraw from the study will not affect your relationship with UNSW Sydney. If you decide to leave the research study, the researchers will not collect additional information from you. You can request that any identifiable information about you be withdrawn from the research project.

1. **What if I have a complaint or any concerns about the research study?**

If you have a complaint regarding any aspect of the study or the way it is being conducted, please contact the UNSW Human Ethics Coordinator:

**Complaints Contact**

| **Position** | UNSW Human Research Ethics Coordinator |
| --- | --- |
| **Telephone** | + 61 2 9385 6222 |
| **Email** | [humanethics@unsw.edu.au](mailto:humanethics@unsw.edu.au) |
| **HC Reference Number** | HC210400 |

1. **What should I do if I have further questions about my involvement in the research study?**

The person you may need to contact will depend on the nature of your query. If you require further information regarding this study or if you have any problems which may be related to your involvement in the study, you can contact the following member/s of the research team:

**Research Team Contact Details**

| **Name** | Dr Lauren McGillivray – trial manager |
| --- | --- |
| **Position** | Post-doctoral Research Fellow |
| **Telephone** | (02) 9382 9289 |
| **Email** | [pts@blackdog.org.au](mailto:pts@blackdog.org.au) |

**Chief Investigator**

| **Name** | Dr Michelle Tye |
| --- | --- |
| **Position** | Senior Research Fellow |
| **Telephone** | (02) 9382 9289 |
| **Email** | [m.torok@unsw.edu.au](mailto:m.torok@unsw.edu.au) |

**Support Services Contact Details**

If at any stage during the study, you become distressed or require additional support from someone not involved in the research please call Lifeline (13 11 14).

**Consent Form – Participant providing own consent**

**Declaration by the participant**

- I understand I am being asked to provide consent to participate in this research study;
- I have read the Participant Information Sheet, or someone has read it to me in a language that I understand;
- I understand the purposes, study tasks and risks of the research described in the study;
- I understand and agree that my activity on the App will be tracked and accessed by the research team (e.g., number of modules accessed).
- Recordings: I understand that the research team will audio record the interview; I agree to be recorded for this purpose.
- I provide my consent for the information collected about me to be used for the purpose of this research study only.
- I have had an opportunity to ask questions and I am satisfied with the answers I have received;
- I freely agree to participate in this research study as described and understand that I am free to withdraw at any time during the study and withdrawal will not affect my relationship with any of the named organisations and/or research team members;
- I understand that I will be given a signed copy of this document to keep.
- I would like to receive a copy of the study results via email or post, I have provided my details below and ask that they be used for this purpose only.
- *Check all of the above.*

**Name: _____________________________________**

**Email Address: ______________________________**

**Phone:**

**Optional Consent for reuse of data and future research:**

- I provide my consent for the information collected about me to made available to other researchers as described at section 6 of this document.

**Gillick Competency Task**

This study will include participants aged 17 years to be able to consent themselves into this study as mature minors. To ensure that these young people are fully aware of the study’s intent, terms, and consequences, they will be asked to complete the following 5-item Gillick Competency Task. If they fail this task (by answering any of the questions incorrectly), they will not be able to complete the survey and will be offered a message saying they are unable to successfully consent to this study and provided a list of help resources for mental health and suicide.

| What the participant will see | | Drop down response options available |
| --- | --- | --- |
| What is this study about? | --- select --- | To find out if a mental health app - LifeBuoy - is effective in helping young people manage their suicidal thoughts (1)  Evaluating if an online game can prevent metal disorder (0)  I have no idea (0) |
| How old do you have to be to participate in this study? | --- select --- | Any age (0)  17-24 (1)  I don’t know (0) |
| What does the study involve? | --- select --- | I will be allocated to use an app and complete four questionnaires over 120 days (1)  I will need to complete a series of online questionnaires to win an Apple iPhone 12 Pro (0)  I don’t know (0) |
| How many questionnaires will you be asked to complete? | --- select --- | Four (1)  One (0)  I don’t know (0) |
| Do you feel pressured to participate in this study? | --- select --- | Yes (0)  No (1)  I’m not sure (0) |
| **Scoring:** sum the five items and a total score of 5 is eligible. A total ≠ 5 is ineligible. | | |

**Form for Withdrawal of Participation**

I wish to **WITHDRAW** my consent to participate in this research study described above and understand that such withdrawal **WILL NOT** affect my relationship with The University of New South Wales.

- I am withdrawing my consent and I would like any identifiable information collected about me which I have provided for the purpose of this research study withdrawn.
- I am withdrawing my consent to participate in further components of this research and provide my permission for the research team to retain and/or use information collected about me which I have provided for the purpose of this research.

**Participant Name**

| Name of Participant (please type) |  |
| --- | --- |
| Date |  |

**The section for Withdrawal of Participation should be forwarded to:**

| CI Name: | Dr Michelle Tye |
| --- | --- |
| Email: | [m.torok@unsw.edu.au](mailto:m.torok@unsw.edu.au) |
| Phone: | (02) 9382 9289 |
| Postal Address: | Black Dog Institute, Hospital Road, NSW 2031 |

**Screening eligibility survey**

Hi, and thanks for your interest in our study! To get started, we just need to ask you a few questions to see if you are eligible to participate. The questions should only take 1 to 2 minutes, and all your answers are confidential.

| What the participant will see | | Drop down response options available |
| --- | --- | --- |
| How old are you? | --- select --- | under 17 years (0)  17 years (1)  18-24 years (1)  25 years or older (0) |
| Are you currently living in Australia? | --- select --- | No (0)  Yes (1) |
| Will you be living in Australia for the next 6 months? | --- select --- | No (0)  Yes (1) |
| Are you fluent in English? | --- select --- | No (0)  Yes (1) |
| Do you own or have access to a smartphone (iOS or Android)? | --- select --- | No (0)  Yes (1) |
| Have you experienced suicidal thoughts in the past 1-month (these can include passive thoughts e.g., “I wish I was dead”)? | --- select --- | No (0)  Yes (1) |
| Have you seen a mental health professional for a mental health concern in the past 6 months? | --- select --- | No (0)  Yes (0) |
| Have been diagnosed with psychosis or bipolar disorder in the past 30 days? | --- select --- | No (1)  Yes (0) |
| **Scoring**: sum 8 items. Eligible if score = 7 only. | | |

(if participant selects age 17 years)

| What the participant will see | | Drop down response options available |
| --- | --- | --- |
| What is this study about? | --- select --- | To find out if a mental health app - LifeBuoy - is effective in helping young people manage their suicidal thoughts (1)  Evaluating if an online game can prevent metal disorder (0)  I have no idea (0) |
| How old do you have to be to participate in this study? | --- select --- | Any age (0)  17-24 (1)  I don’t know (0) |
| What does the study involve? | --- select --- | I will be allocated to one of three groups (intervention app, intervention app + engagement, or control app), use the app I am allocated, and complete four questionnaires over 4-months (1)  I will need to complete a series of online questionnaires to win an Apple iPhone 12 Pro (0)  I don’t know (0) |
| How many questionnaires will you be asked to complete? | --- select --- | Four (1)  One (0)  I don’t know (0) |
| Do you feel pressured to participate in this study? | --- select --- | Yes (0)  No (1)  I’m not sure (0) |
| **Sum the five items.** If total score = 5 then they are eligible. If total ≠ 5 then ineligible. | | |

(If eligible)

Ok great, thank you so much for taking the time to answer these questions. Based on your responses, it looks like you are:

Eligible

This is great news! If you are willing to take part in the study, we will need to click through to the next page and register before completing your first assessment and downloading the app.

(If ineligible)

Thank you for your interest in our study. Based on your responses, it looks like you are:

Not eligible

Your responses indicate that you are unable to participate in this study because you don’t meet the selection criteria. Your willingness to participate in this study is hugely appreciated. And just because this might not be the right study for you, or the right time for you to participate, it does not mean that you cannot participate in other studies.

Also know that you are not alone. If you’ve had a tough time with your mental health lately, we encourage you to talk to a local doctor or mental health professional about how you’re feeling. If you would like to know where to get some immediate help, here are some suggestions:

Lifeline: 13 11 14

Kids Helpline: 1800 55 1800

Suicide Callback Service: 1300 659 467

For 24 hour advice on what to do and who to contact, don’t hesitate to visit HealthDirect (https:/[/www.h](http://www.healthdirect.gov.au/)e[althdirect.gov.au](http://www.healthdirect.gov.au/) ) or call them on 1800 022 222. They can help you figure out the best people to contact in your local area. If you feel in danger or that you might hurt yourself, do not hesitate to call Emergency Services on 000.

**Outcome measurement surveys**

Demographics – baseline only

| What the participant will see | | Drop down response options available |
| --- | --- | --- |
| Age | --- select ---- | 17  18  19  20  21  22  23  24 |
| Gender identity | --- select ---- | Female (1)  Male (2)  Non-binary (3)  Prefer not to answer (4) |
| Gender assigned at birth | --- select ---- | Female (1)  Male (2) |
| Which state or territory do you live in? | -- select ---- | NSW (1)  QLD (2)  VIC (3)  TAS (4)  SA (5)  WA (6)  NT (7)  ACT (8) |
| Do you live in rural/remote or metropolitan area? | -- select ---- | Metropolitan (1)  Rural and/or remote (2) |
| Do you identify as Lesbian, Gay, Bisexual, Queer, or anything other than heterosexual? | -- select ---- | No (0)  Yes (1)  Prefer not to say (2) |
| Language/s spoken at home | -- select ---- | English only (1)  English and other language (2)  Another language only (3) |
| Current living situation | -- select ---- | Live on my own, alone (1)  Live with parents (2)  Live with other family (3)  Live with a significant other (4)  Live with flatmate(s) or friends (5)  Other (6) |
| Current relationship situation | -- select ---- | Not in a relationship (0)  Partner/dating (1)  De facto/married (2)  Separated (3)  Divorced or widowed (4)  Other (5) |
| Highest level of education completed | -- select ---- | school qualification (1)  certificate level (2)  advanced diploma & diploma (3)  bachelor degree (4)  Postgraduate degree (5) |
| What is your main current employment status? | -- select ---- | Unemployed (0)  Student – high school (1)  Student – university (2)  Casual (3)  Part-time (4)  Full-time (5) |
| Have you ever experienced mental illness or been diagnosed with mental illness? | -- select ---- | No (0)  Yes – I’ve experienced it but never been diagnosed (1)  Yes – I have been diagnosed (2)  I don’t know (3) |
| Have you ever seen a mental health professional (psychologist, psychiatrist) for a mental health problem? | --- select ---- | No (0)  Yes (1)  I don’t know (2) |
| (skip if No (0) to above question)  How many independent/separate mental health professionals have you seen in your lifetime? | --- select ---- | 1  2  3  4  5+ |
| Are you currently receiving treatment for a mental health issue (i.e., medication or therapy)? | --- select ---- | No (0)  Yes, medication only (1)  Yes, therapy only (2)  Yes, both (3) |
| (If 2 or 3) How many sessions have you had with your current mental health professional? | (free numerical response) | 0 to 1000 |
| What is the longest you have stayed with a mental health professional | --- select ---- | N/A  Less than a month (1)  1 to 3 months (2)  3 to 6 months (3)  6 to 12 months (4)  1 to 2 years (5)  More than 2 years (6) |

Now we’re going to ask you some questions about suicidal thoughts, and the severity of them. Please think about the past 30-days when answering the following questions.

[Suicidal Ideation Attributes Scale] (SIDAS)

| Question | Response options |
| --- | --- |
| In the past month, how often have you had thoughts about suicide? | 0 (Never), 1,2,3,4,5,6,7,8,9, 10 (Always) |
| In the past month, how much control have you had over these thoughts? | 10 (Full control), 9, 8, 7, 6, 5, 4, 3, 2, 1, 0 (No control) |
| In the past month, how close have you come to making a suicide attempt? | 0 (Not close at all), 1,2,3,4,5,6,7,8,9, 10 (Made an attempt) |
| In the past month, to what extent have you felt tormented by thoughts about suicide? | 0 (Not at all), 1,2,3,4,5,6,7,8,9, 10 (Extremely) |
| In the past month, how much have thoughts about suicide interfered with your ability to carry out daily activities, such as work, household tasks or social activities? | 0 (Not at all), 1,2,3,4,5,6,7,8,9, 10 (Extremely) |
| **Note:**Respondents who respond “0 – Never” to the first item skip all remaining items and score a total of zero. **Note:** item 2 is reverse scored.  **Scoring:** add total (range from 0 to 50) | |

We’d just like to ask you a few questions about prior self-harm thoughts and behaviours. Please look after yourself as you go through these questions and take breaks as needed.

[Other suicide-related questions] (Other_SI)

| What the user will see | | Drop down response options available |
| --- | --- | --- |
| How many times have you made an attempt to kill yourself in which you had some intent to die?  *Baseline only* | (free numerical response) | Participant to type in the number |
| (If >0) How many suicide attempts were made in the past 12 months?  *Baseline only* | (free numerical response) | Participant to type in the number |
| (If >0) How many suicide attempts were made in the past 30 days? | (free numerical response) | Participant to type in the number |
| (If yes to the past 30 days)  In the most severe suicide attempt in the past 30 days: | - Select all that apply - | No care was needed (0)  I attended the hospital, but without physical injury and left without being admitted (1)  I attended the hospital with a physical injury but left without being admitted (2)  I was admitted to hospital without physical injury (3)  I was admitted to hospital with physical injury (4) |
| How long have you been experiencing suicidal thoughts?  *Baseline only* | --- select ---- | 3 months or less (1)  Up to 12 months (2)  More than 12 months (3)  More than two years (4)  More than five years (5) |
| At what age did you first experience suicidal thoughts?  *Baseline only* | (free numerical response) | Participant to type in the number |
| Have you ever injured yourself on purpose *without suicidal intent* (i.e., intentional, self-inflicted damage to the surface of the body *with no intent to die*)  *Baseline only* | --- select ---- | No (0)  Yes (1) |
| (If yes) How many times have you injured yourself on purpose in the past 12 months?  *Baseline only* | (free numerical response) | Participant to type in the number |
| (If >0) How many times have you injured yourself on purpose in the past 30 days? | (free numerical response) | Participant to type in the number |
| (If past 30 days more than 0) In the most severe self-injury over the past 30-days (please select one): | --- select ---- | No medical care was needed (0),  I treated the injury myself (1),  I attended a medical clinic or hospital and left without being admitted (2),  I was admitted to hospital (3) |

[Patient Health Questionnaire-9] (PHQ-9)

| Over the last **2 weeks**, how often have you been bothered by the following problems? | Not at all | Several days | More than half the days | Nearly every day |
| --- | --- | --- | --- | --- |
| Little interest or pleasure in doing things | 0 | 1 | 2 | 3 |
| Feeling down, depressed, irritable, or hopeless | 0 | 1 | 2 | 3 |
| Trouble falling asleep, staying asleep, or sleeping too much | 0 | 1 | 2 | 3 |
| Feeling tired, or having little energy | 0 | 1 | 2 | 3 |
| Poor appetite, weight loss, or overeating | 0 | 1 | 2 | 3 |
| Feeling bad about yourself — or feeling that you are a failure, or that you have let yourself or your family down | 0 | 1 | 2 | 3 |
| Trouble concentrating on things, such as reading the newspaper or watching television | 0 | 1 | 2 | 3 |
| Moving or speaking so slowly that other people could have noticed? Or the opposite — being so fidgety or restless that you were moving around a lot more than usual | 0 | 1 | 2 | 3 |
| Thoughts that you would be better off dead, or of  hurting yourself in some way | 0 | 1 | 2 | 3 |
| **Scoring:** add total (range from 0 to 27) **Severity:** total scores of 5, 10, 15, and 20 indicate mild, moderate, moderately-severe, and severe depressive symptoms. | | | | |

[Generalised Anxiety Disorder-7] (GAD-7)

| Over the **last 2 weeks**, how often have you been bothered by the following problems? | Not at all | Several days | More than half the days | Nearly every day |
| --- | --- | --- | --- | --- |
| Feeling nervous, anxious, or on edge | 0 | 1 | 2 | 3 |
| Not being able to stop or control worrying | 0 | 1 | 2 | 3 |
| Worrying too much about different things | 0 | 1 | 2 | 3 |
| Trouble relaxing | 0 | 1 | 2 | 3 |
| Being so restless that it's hard to sit still | 0 | 1 | 2 | 3 |
| Becoming easily annoyed or irritable | 0 | 1 | 2 | 3 |
| Feeling afraid as if something awful might  happen | 0 | 1 | 2 | 3 |
| **Scoring:** add total (range from 0 to 21)  **Severity:** total scores of 5, 10, 15 indicate mild, moderate, and severe generalised anziety symptoms. | | | | |

[Social Phobia Inventory] (mini-SPIN)

| Please indicate how much the following problems have bothered you during the past week. | | Not at all | A little bit | Somewhat | Very much | Extremely |
| --- | --- | --- | --- | --- | --- | --- |
| Fear of embarrassment causes me to avoid doing things or speaking to people | | 0 | 1 | 2 | 3 | 4 |
| I avoid activities in which I am the centre of attention | | 0 | 1 | 2 | 3 | 4 |
| Being embarrassed or looking stupid is among my worst fears | | 0 | 1 | 2 | 3 | 4 |
| **Scoring:** add total (range from 0 to 12)  **Severity**: total score of 6 or more indicates presence of social phobia. | | | | | | |

[The Tobacco, Alcohol, Prescription medications, and other Substance] (TAPS-1)

| On average, in the past 30 days, how often have you: | I didn’t use | 1-2 times a fortnight | 1-3 times a week | More than 4 times a week | Daily |
| --- | --- | --- | --- | --- | --- |
| Used any tobacco product (for example, cigarettes, e- cigarettes, cigars, pipes, or smokeless tobacco) | 1 | 2 | 3 | 4 | 5 |
| Had 4 or more drinks containing alcohol in one day (one standard drink is 1x small glass of wine, 1x beer, or 1x single shot of liquor) | 1 | 2 | 3 | 4 | 5 |
| Used any illicit drugs including marijuana, cocaine or crack, heroin, methamphetamine (crystal meth), hallucinogens, ecstasy/MDMA | 1 | 2 | 3 | 4 | 5 |
| Used any prescription medications just for the feeling, more than prescribed or that were not prescribed for you (e.g., opiate pain relievers, medications for anxiety or sleeping, or for ADHD) | 1 | 2 | 3 | 4 | 5 |
| **Scoring:** add total (range from 4 to 20) | | | | | |

[AOD use and suicidal ideation] (AOD_SI)

| What the user will see | | Drop down response options available |
| --- | --- | --- |
| Have you ever used alcohol to help you cope with suicidal thoughts? | --- select ---- | No (0)  Yes (1) |
| Have you ever used cigarettes to help you cope with suicidal thoughts? | --- select ---- | No (0)  Yes (1) |
| Have you ever used illicit drugs to help you cope with suicidal thoughts? | --- select ---- | No (0)  Yes (1) |

[The Barratt Impulsiveness Scale] (BIS-Brief)

| People differ in the ways they act and think in different situations. This is a test to measure some of the ways in which you act and think. Read and rate each statement. | Rarely/Never | Occasionally | Often | Almost always/ always |
| --- | --- | --- | --- | --- |
| I plan tasks carefully. | 4 | 3 | 2 | 1 |
| I do things without thinking. | 1 | 2 | 3 | 4 |
| I don’t pay attention. | 1 | 2 | 3 | 4 |
| I am self-controlled. | 4 | 3 | 2 | 1 |
| I concentrate easily. | 4 | 3 | 2 | 1 |
| I am a careful thinker. | 4 | 3 | 2 | 1 |
| I say things without thinking. | 1 | 2 | 3 | 4 |
| I act on the spur of the moment. | 1 | 2 | 3 | 4 |
| **Note:** items 1, 4, 5, & 6 have been reverse-scored.  **Scoring:** add total (range from 4 to 32) | | | | |

Now we’d like to ask you some questions about social relationships and stressors to understand what’s going on in your life.

[Brief Daily Stressors Screening Tool] (BDSS)

There are occasional minor and major challenges in daily life that can constantly reoccur, to which one can sometimes not get used to, and which can be more or less burdensome. Please select whether and how strongly you have been affected by the following annoyances or inconveniences *over the past 30-days*.

| My exposure to: | Not at all |  |  |  | Very much |
| --- | --- | --- | --- | --- | --- |
| Difficulties with social obligations (e.g., associations, organizations) | 0 | 1 | 2 | 3 | 4 |
| Difficulties with family responsibilities (e.g., household, care services, parenting, school) | 0 | 1 | 2 | 3 | 4 |
| Health problems (e.g., diseases, chronic sufferings) | 0 | 1 | 2 | 3 | 4 |
| Financial restrictions (e.g. low income, installments) | 0 | 1 | 2 | 3 | 4 |
| Dissatisfaction with education/occupation (e.g. examinations, work overload) | 0 | 1 | 2 | 3 | 4 |
| Difficulties with (secondary) employment (e.g. compatibility with school/college, high responsibility, noise pollution) | 0 | 1 | 2 | 3 | 4 |
| Dissatisfaction with housing situation (e.g., noise, small flat) | 0 | 1 | 2 | 3 | 4 |
| Frequent contradictions, conflicts, tensions with: | | | | | |
| Close friends or family (e.g., family, household, friends) | 0 | 1 | 2 | 3 | 4 |
| Romantic partner (e.g., someone you’re dating, in a romantic relationship with, or married to) | 0 | 1 | 2 | 3 | 4 |
| Other persons (e.g., colleagues, fellow - students, neighbors, tenants, landlords) | 0 | 1 | 2 | 3 | 4 |
| Another burden/stressor not yet mentioned | 0 | 1 | 2 | 3 | 4 |
| **Scoring:** add total (range from 0 to 44) | | | | | |

[Expected app use and motivation] (Use_Exp)

| What the participant will see | | Drop down response options available |
| --- | --- | --- |
| How satisfied have you been with face-to-face treatment overall |  | N/A (0)  Very satisfied (5)  Satisfied (4)  Neither satisfied nor dissatisfied (3)  Dissatisfied (2)  Very dissatisfied (1) |
|  | --- select ---- |  |
|  |  |  |
| Are you looking for alternative mental health treatment to face-to-face therapy? | --- select ---- | No (0)  Yes (1) |
| (If yes) Why? | (free text) | Participant to type in a response |
| If you are currently in face-to-face treatment, how are you expecting to use this app? | --- select all that apply ---- | N/A (0)  To fill a gap between sessions (1)  During face-to-face sessions (2)  To draw out time between face-to-face sessions (3)  When I cannot access my therapist (4)  Other <free text> |
| What motivated you to participate in this study? | (free text) | Participant to type in a response |

[Mobile Application Rating Scale - user version] (uMARS) – T1/30-day assessment only

| We'd like to see what you thought about LifeBuoy. This will help us improve the app. Please select the answer that most accurately represents the quality of the LifeBuoy app.  [quality ratings] | | Drop down response options available |
| --- | --- | --- |
| Entertainment: Is the app fun/entertaining to use? Does it have components that make it  more fun than other similar apps? | --- select ---- | 1 - Dull, not fun or entertaining at all  2 - Mostly boring  3 - OK, fun enough to entertain user for a brief time (less than 5 minutes)  4 - Moderately fun and entertaining, would entertain user for some time (5-10 minutes total)  5 - Highly entertaining and fun, would stimulate repeat use |
| Interest: Is the app interesting to use? Does it present its information in an interesting way  compared to other similar apps? | --- select ---- | 1 - Not interesting at all  2 - Mostly uninteresting  3 - OK, neither interesting nor uninteresting; would engage user for a brief time (less than 5 minutes)  4 - Moderately interesting; would engage user for some time (5-10 minutes total)  5 - Very interesting, would engage user in repeat use |
| Customisation: Does it allow you to customise the settings and preferences that you would  like to (e.g. sound, content and notifications)? | --- select ---- | 1 - Does not allow any customisation or requires setting to be input every time  2 - Allows little customisation and that limits app’s functions  3 - Basic customisation to function adequately  4 - Allows numerous options for customisation  5 - Allows complete tailoring the user’s characteristics/preferences, remembers all settings |
| Interactivity: Does it allow user input, provide feedback, contain prompts (reminders, sharing  options, notifications, etc.)? | --- select ---- | 1 - No interactive features and/or no response to user input  2 - Some, but not enough interactive features which limits app’s functions  3 - Basic interactive features to function adequately  4 - Offers a variety of interactive features, feedback and user input options  5 - Very high level of responsiveness through interactive features, feedback and user input options |
| Target group: Is the app content (visuals, language, design) appropriate for the target  audience? | --- select ---- | 1 - Completely inappropriate, unclear or confusing  2 - Mostly inappropriate, unclear or confusing  3 - Acceptable but not specifically designed for the target audience. May be inappropriate/  unclear/confusing at times  4 - Designed for the target audience, with minor issues  5 - Designed specifically for the target audience, no issues found |
| Performance: How accurately/fast do the app features (functions) and components  (buttons/menus) work? | --- select ---- | 1 - App is broken; no/insufficient/inaccurate response (e.g. crashes/bugs/broken features, etc.)  2 - Some functions work, but lagging or contains major technical problems  3 - App works overall. Some technical problems need fixing, or is slow at times  4 - Mostly functional with minor/negligible problems  5 - Perfect/timely response; no technical bugs found, or contains a ‘loading time left’ indicator (if  relevant) |
| Ease of use: How easy is it to learn how to use the app; how clear are the menu labels, icons  and instructions? | --- select ---- | 1 - No/limited instructions; menu labels, icons are confusing; complicated  2 - Takes a lot of time or effort  3 - Takes some time or effort  4 - Easy to learn (or has clear instructions)  5 - Able to use app immediately; intuitive; simple (no instructions needed) |
| Navigation: Does moving between screens make sense; Does app have all necessary links  between screens? | --- select ---- | 1 - No logical connection between screens at all /navigation is difficult  2 - Understandable after a lot of time/effort  3 - Understandable after some time/effort  4 - Easy to understand/navigate  5 - Perfectly logical, easy, clear and intuitive screen flow throughout, and/or has shortcuts |
| Gestural design: Do taps/swipes/pinches/scrolls make sense? Are they consistent across all  components/screens? | --- select ---- | 1 - Completely inconsistent/confusing  2 - Often inconsistent/confusing  3 - OK with some inconsistencies/confusing elements  4 - Mostly consistent/intuitive with negligible problems  5 - Perfectly consistent and intuitive |
| Layout: Is arrangement and size of buttons, icons, menus and content on the screen  appropriate? | --- select ---- | 1 - Very bad design, cluttered, some options impossible to select, locate, see or read  2 - Bad design, random, unclear, some options difficult to select/locate/see/read  3 - Satisfactory, few problems with selecting/locating/seeing/reading items  4 - Mostly clear, able to select/locate/see/read items  5 - Professional, simple, clear, orderly, logically organised |
| Graphics: How high is the quality/resolution of graphics used for buttons, icons, menus and  content? | --- select ---- | 1 - Graphics appear amateur, very poor visual design - disproportionate, stylistically inconsistent  2 - Low quality/low resolution graphics; low quality visual design – disproportionate  3 - Moderate quality graphics and visual design (generally consistent in style)  4 - High quality/resolution graphics and visual design – mostly proportionate, consistent in style  5 - Very high quality/resolution graphics and visual design - proportionate, consistent in style  throughout |
| Visual appeal: How good does the app look? | --- select ---- | 1 - Ugly, unpleasant to look at, poorly designed, clashing, mismatched colours  2 - Bad – poorly designed, bad use of colour, visually boring  3 - OK – average, neither pleasant, nor unpleasant  4 - Pleasant – seamless graphics – consistent and professionally designed  5 - Beautiful – very attractive, memorable, stands out; use of colour enhances app features/menus |
| Quality of information: Is app content correct, well written, and relevant to the goal/topic of  the app? | --- select ---- | N/A - There is no information within the app  1 - Irrelevant/ inappropriate/ incoherent/ incorrect  2 - Poor. Barely relevant/ appropriate/ coherent/ may be incorrect  3 - Moderately relevant/ appropriate/ coherent/ and appears correct  4 - Relevant/ appropriate/ coherent/ correct  5 - Highly relevant, appropriate, coherent, and correct |
| Quantity of information: Is the information within the app comprehensive but concise? | --- select ---- | N/A - There is no information within the app  1 - Minimal or overwhelming  2 - Insufficient or possibly overwhelming  3 - OK but not comprehensive or concise  4 - Offers a broad range of information, has some gaps or unnecessary detail; or has no links to  more information and resources  5 - Comprehensive and concise; contains links to more information and resources |
| Visual information: Is visual explanation of concepts – through charts/graphs/images/videos,  etc. – clear, logical, correct? | --- select ---- | N/A - There is no visual information within the app (e.g. it only contains audio, or text)  1 - Completely unclear/confusing/wrong or necessary but missing  2 - Mostly unclear/confusing/wrong  3 - OK but often unclear/confusing/wrong  4 - Mostly clear/logical/correct with negligible issues  5 - Perfectly clear/logical/correct |
| Credibility of source: does the information within the app seem to come from a credible  source? | --- select ---- | N/A - There is no information within the app  1 - Suspicious source  2 - Lacks credibility  3 - Not suspicious but legitimacy of source is unclear  4 - Possibly comes from a legitimate source  5 - Definitely comes from a legitimate/specialised source |
| [subjective quality] | | |
| Would you recommend this app to people who might benefit from it? | --- select ---- | 1 - Not at all. I would not recommend this app to anyone  2 - There are very few people I would recommend this app to  3 – Maybe. There are several people I would recommend this app to  4 - There are many people I would recommend this app to  5 – Definitely. I would recommend this app to everyone |
| How many times do you think you would use this app in the next 12 months if it was relevant  to you? | --- select ---- | 1 - None  2 - 1 to 2  3 - 3 to 10  4 - 10 to 50  5 – more than 50 |
| Would you pay for this app? | --- select ---- | 1 - Definitely not  2 - Probably not  3 - Maybe  4 - I might  5 - Definitely yes |
| What is your overall (star) rating of the app? | --- select ---- | 1 – One star. One of the worst apps I’ve used  2 - Two stars  3 - Three stars. Average  4 - Four stars  5 - Five stars. One of the best apps I've used |
| [perceived impact] | | |
| Awareness – This app has increased my awareness of the importance of addressing my suicidal ideation | 1= Strongly disagree, 2, 3, 4, 5= Strongly agree | |
| Knowledge – This app has increased my knowledge/understanding of my suicidal ideation | 1= Strongly disagree, 2, 3, 4, 5= Strongly agree | |
| Attitudes – The app has changed my attitudes toward improving my suicidal ideation | 1= Strongly disagree, 2, 3, 4, 5= Strongly agree | |
| Intention to change – The app has increased my intentions/motivation to address my suicidal ideation | 1= Strongly disagree, 2, 3, 4, 5= Strongly agree | |
| Help seeking – This app would encourage me to seek further help to address my suicidal ideation (if I needed it) | 1= Strongly disagree, 2, 3, 4, 5= Strongly agree | |
| Behaviour change – Use of this app will decrease my suicidal ideation | 1= Strongly disagree, 2, 3, 4, 5= Strongly agree | |
| Further comments about the app? | <free-text response option> | |
| **Note**: ‘Subjective quality’ and ‘perceived impact’ are not scored.  **Scoring:** sum scores and divide by 4 for overall ‘app quality’ mean score. **Subfactors:** items 1-5 = engagement; 6-9 = functionality; 10-12 = aesthetics; and 13-16 = information | | |

(if participant was assigned to the “LifeBuoy + engagement strategy” arm)

We’d like to hear your thoughts about the Instagram posts. There are no right or wrong answers. Please answer as honestly as you can.

[Engagement Strategy] (Engage) – Assessed at T1, T2, T3 in LifeBuoy+Eng arm only

| Question | Response options | | |
| --- | --- | --- | --- |
| Please provide an estimate of the total number of Instagram posts that you viewed during the study. | Free numeric response | | |
| The posts were visually appealing. | 0=Strongly  Disagree | 1,2,3,4,5,6,7,8,9 | 10=Strongly  Agree |
| The posts were informative. | 0=Strongly  Disagree | 1,2,3,4,5,6,7,8,9 | 10=Strongly  Agree |
| The posts were easy to understand. | 0=Strongly  Disagree | 1,2,3,4,5,6,7,8,9 | 10=Strongly  Agree |
| The posts provided information that was easy for me to apply to my own situation. | 0=Strongly  Disagree | 1,2,3,4,5,6,7,8,9 | 10=Strongly  Agree |
| In general, posts were interesting and engaging. | 0=Strongly  Disagree | 1,2,3,4,5,6,7,8,9 | 10=Strongly  Agree |
| The posts helped remind me to use LifeBuoy. | 0=Strongly  Disagree | 1,2,3,4,5,6,7,8,9 | 10=Strongly  Agree |
| The posts made me more interested to use LifeBuoy. | 0=Strongly  Disagree | 1,2,3,4,5,6,7,8,9 | 10=Strongly  Agree |
| The posts made me more confident that I would find Lifebuoy helpful. | 0=Strongly  Disagree | 1,2,3,4,5,6,7,8,9 | 10=Strongly  Agree |
| Email notifications informing me of Instagram updates were irritating. | 10=Strongly  Disagree | 9,8,7,6,5,4,3,2,1 | 0=Strongly  Agree |
| Overall, I think the Instagram posts helped me engage more with LifeBuoy. | 0=Strongly  Disagree | 1,2,3,4,5,6,7,8,9 | 10=Strongly  Agree |
| Which aspect(s) of the Instagram posts did you like, and why? |  | Free text response |  |
| Which aspect(s) of the Instagram posts did you not like, and why? |  | Free text response |  |
| If you felt that the Instagram posts helped you engage more with LifeBuoy, please tell us how you think the posts contributed to your decision to use LifeBuoy more. |  | Free text response |  |
| **Note:** one item has been reverse scored. | | | |

We’d like to hear your thoughts about the blog. There are no right or wrong answers. Please answer as honestly as you can

| Question | Response options | | |
| --- | --- | --- | --- |
| Please provide an estimate of the total number of blog articles that you viewed during the study. | Free numeric response | | |
| The blog webpage were visually appealing. | 0=Strongly  Disagree | 1,2,3,4,5,6,7,8,9 | 10=Strongly  Agree |
| The articles were informative. | 0=Strongly  Disagree | 1,2,3,4,5,6,7,8,9 | 10=Strongly  Agree |
| The articles were easy to understand. | 0=Strongly  Disagree | 1,2,3,4,5,6,7,8,9 | 10=Strongly  Agree |
| The articles provided information that was easy for me to apply to my own situation. | 0=Strongly  Disagree | 1,2,3,4,5,6,7,8,9 | 10=Strongly  Agree |
| In general, articles were interesting and engaging. | 0=Strongly  Disagree | 1,2,3,4,5,6,7,8,9 | 10=Strongly  Agree |
| The articles helped remind me to use LifeBuoy. | 0=Strongly  Disagree | 1,2,3,4,5,6,7,8,9 | 10=Strongly  Agree |
| The articles made me more interested to use LifeBuoy. | 0=Strongly  Disagree | 1,2,3,4,5,6,7,8,9 | 10=Strongly  Agree |
| The articles made me more confident that I would find Lifebuoy helpful. | 0=Strongly  Disagree | 1,2,3,4,5,6,7,8,9 | 10=Strongly  Agree |
| Email notifications informing me of blog updates were irritating. | 10=Strongly  Disagree | 9,8,7,6,5,4,3,2,1 | 0=Strongly  Agree |
| Overall, I think the blog helped me engage more with LifeBuoy. | 0=Strongly  Disagree | 1,2,3,4,5,6,7,8,9 | 10=Strongly  Agree |
| Which aspect(s) of the blog did you like, and why? |  | Free text response |  |
| Which aspect(s) of the blog did you not like, and why? |  | Free text response |  |
| If you felt that the blog helped you engage more with LifeBuoy, please tell us how you think the blog contributed to your decision to use LifeBuoy more. |  | Free text response |  |
| **Note:** one item has been reverse scored. | | | |

**Fortnightly 2-item survey for LifeBuoy+engagement arm only**

Hi, thanks for taking the time to answer these two questions!

| What the participant will see | | Response options |
| --- | --- | --- |
| Have you visited the Instagram page in the last 2-weeks? | --- select --- | No (0)  Yes (1) |
| Have you visited the Blog website in the last 2-weeks? | --- select --- | No (0)  Yes (1) |

# **Risk Management Protocol**

**Estimated flag and call-back rates**

Trial one, LifeBuoy study: 455 participants completed baseline; 259 were flagged (i.e., scored SIDAS >20); and 5/259 (1.93%) requested a call back from the team clinical psychologist.

**Flagging procedure**

**1)** At each survey timepoint (baseline, 30-days, 60-days, and 120-days) three items indicates risk of suicide. When a selection is made indicating a concerning level of suicidal thinking (SIDAS score >20), a suicide attempt during the trial, or NSSI requiring medical care during the trial, an automated alert system is triggered, where an automated email is sent to the participant asking if they would like to receive a phone call from the team clinical psychologist during business hours (9:00 to 17:00 AEST) within the next 3 working days. This email will also include a list of crisis contact numbers for support.

*“Hi there,*

*Thanks so much for signing up to participate in the study and for completing the latest survey. We noticed that your responses on the survey indicate a recent self-harm event or severe current suicidal thoughts. If you would like to receive a phone call from our clinical psychologist during business hours (9:00 to 17:00 AEST) within the next 3 working days, please click ‘reply’ to this email or send a new email request to pts@blackdog.org.au*

*If you’ve had a tough time with your mental health lately, we encourage you to talk to a local doctor or mental health professional about how you’re feeling. If you would like to know where to get some immediate help, here are some suggestions:*

- *Lifeline: 13 11 14*
- *Kids Helpline :1800 55 1800*
- *Suicide Callback Service: 1300 659 467*

*For 24-hour advice on what to do and who to contact, don’t hesitate to visit HealthDirect (https://www.healthdirect.gov.au) or call them at 1800 022 222).*

*If you feel in danger or that you might hurt yourself, do not hesitate to call Emergency Services on 000.*

*Warm regards*

*The LifeBuoy team”*

(*Note:* at the completion of each survey all participants will be shown a screen with the same abovementioned support contacts).

**2)** If a flagged participant indicates they would like a call back, the team clinical psychologist (Dr Lauren McGillivray) will contact the participant via phone as soon as possible. This discussion will centre around making sure the young person is supported in their family and/or community and identifying how they can access the services they need. If the participant expresses distress in relation to using the app or answering the assessment questionnaires, they will be reminded of their right to withdraw from the trial without penalty or the need for explanation.

**3)** In the event that a participant does not answer the initial contact attempt, an email stating that contact was attempted and a request for their availability will be sent. The psychologist will attempt to call the participants up to two times in this allocated time. If the participant is still unavailable another email stating that contact was attempted, and containing referral sources, will be sent:

*“Hi <firstname>,*

*The team psychologist called you today between <time> as instructed by yourself, but you were not available. They will try up to two times to reach you – please note their call may show up as ‘unknown’ or ‘private’.*

*If you would like to know where to get some immediate help, here are some suggestions:*

- *Lifeline: 13 11 14*
- *Kids Helpline :1800 55 1800*
- *Suicide Callback Service: 1300 659 467*

If you feel in danger or that you might hurt yourself, do not hesitate to call Emergency Services on 000.”

**4)** Details of these interactions will be recorded in a trial management risk spreadsheet, including failed attempts to contact the participant via telephone.

**Outcomes Monitoring**

There will be two levels of monitoring of risk in this project. The first is the trial steering committee which will consist of the Chief Investigator (Michelle Tye) and research team listed on the ethics application, and the second level is the Data Monitoring Safety Board (DSMB) and this is the group that serious adverse events and reactions will be reported in to. The trial manager will record the number of notifications at each assessment in the Report for DSMB document and will notify the DSMB every 3-months on how many alerts were triggered, and what follow-up was carried out. They will record these in a Case Report spreadsheet (see Appendix B) which will be securely stored on the Institute’s UNSW server.

**Data Storage and Management**

The Black Dog Institute at the University of New South Wales (UNSW) will host the website and store the collected data. All collected data will be held on a secure server at BDI/UNSW and will only be available to researchers and staff working directly on the project.

Some data will be collected directly through the LifeBuoy app, which includes participants’ rating of each challenge and tool, and app usage information (such as time spent in the app, number of lessons accessed and completed). This information gets uploaded to Black Dog Institute servers when the phone is connected to Wi-Fi. Quantitative data will be analysed using SPSS software.
