# Supplementary material for: Adherence and efficacy outcomes in young Australians with suicidal ideation using a self-management app and digital engagement strategy compared with a sham app: a three-arm randomised controlled trial
Source: eClinicalMedicine. 2024 Dec 6;79:102963. doi: 10.1016/j.eclinm.2024.102963 (PMC11665678; doi:10.1016/j.eclinm.2024.102963)
Supplement: CONSORT checklist [file mmc3.docx]

CONSORT Harms 2022 integrated into CONSORT 2010 items checklist of information to include when reporting a randomised trial

| Section/Topic | Item No | Checklist item | Reported on page No |
| --- | --- | --- | --- |
| Title and abstract | | | |
|  | 1a | Identification as a randomised trial in the title | Title |
|  | 1b | Structured summary of trial design, methods, results of outcomes of benefits and harms, and conclusions  (for specific guidance see CONSORT for abstracts) | Abstract |
| Introduction | | | |
| Background and objectives | 2a | Scientific background and explanation of rationale | Introduction, paragraphs 1-3 |
|  | 2b | Specific objectives or hypotheses for outcomes benefits and harms | Introduction, paragraph 4 |
| Methods | | | |
| Trial design | 3a | Description of trial design (such as parallel, factorial) including allocation ratio | Methods, paragraph 2 |
|  | 3b | Important changes to methods after trial commencement (such as eligibility criteria), with reasons | N/A |
| Participants | 4a | Eligibility criteria for participants | Methods, paragraph 2 |
|  | 4b | Settings and locations where the data were collected | Methods, paragraph 2 |
| Interventions | 5 | The interventions for each group with sufficient details to allow replication, including how and when they were actually administered | Methods, paragraphs 4-8 |
| Outcomes | 6a | Completely defined pre-specified primary and secondary outcome measures for both benefits and harms, including how and when they were assessed | Methods, paragraphs 9,11 |
|  | 6b | Any changes to trial outcomes after the trial commenced, with reasons | N/A |
|  | 6c | Describe if and how non-prespecified outcomes of benefits and harms were identified, including any selection  criteria, if applicable | Methods, paragraph 15 |
| Sample size | 7a | How sample size was determined | Methods, paragraph 12 |
|  | 7b | When applicable, explanation of any interim analyses and stopping guidelines | N/A |
| Randomisation: |  |  |  |
| Sequence generation | 8a | Method used to generate the random allocation sequence | Methods, paragraph 3 |
|  | 8b | Type of randomisation; details of any restriction (such as blocking and block size) | Methods, paragraph 3 |
| Allocation concealment mechanism | 9 | Mechanism used to implement the random allocation sequence (such as sequentially numbered containers), describing any steps taken to conceal the sequence until interventions were assigned | Methods, paragraph 3 |
| Implementation | 10 | Who generated the random allocation sequence, who enrolled participants, and who assigned participants to interventions | Methods, paragraph 3 |
| Blinding | 11a | If done, who was blinded after assignment to interventions (e.g., participants, care providers, those assessing outcomes of benefits and harms) and how | Methods, paragraph 3 |
|  | 11b | If relevant, description of the similarity of interventions | N/A |
| Statistical methods | 12a | Statistical methods used to compare groups for primary and secondary outcomes of both benefits and harms | Methods, paragraph 13-15 |
|  | 12b | Methods for additional analyses, such as subgroup analyses and adjusted analyses | Methods, paragraph 16-17 |
| Results | | | |
| Participant flow (a diagram is strongly recommended) | 13a | For each group, the numbers of participants who were randomly assigned, received intended treatment, and were analysed for outcomes of benefits and harms | Results, paragraph 1, Fig 1 |
|  | 13b | For each group, losses and exclusions after randomisation, together with reasons | Fig 1 |
| Recruitment | 14a | Dates defining the periods of recruitment and follow-up for outcomes of benefits and harms | Results, paragraph 1 |
|  | 14b | Why the trial ended or was stopped | N/A |
| Baseline data | 15 | A table showing baseline demographic and clinical characteristics for each group | Results, paragraphs 1-3, Table 1 |
| Numbers analysed | 16 | For each group, number of participants (denominator) included in each analysis and whether the analysis was by original assigned groups and if any exclusions were made | Fig 1 |
| Outcomes and estimation | 17a | For each primary and secondary outcome of benefits and harms, results for each group, and the estimated effect size and its precision (such as 95% confidence interval) | Results, paragraphs 4-13 |
|  | 17a2 | For outcomes omitted from the trial report (benefits and harms), provide rationale for not reporting and indicate where the data on omitted outcomes can be accessed | N/A |
|  | 17b | Presentation of both absolute and relative effect sizes is recommended, for outcomes of benefits and harms | Results, relative effect sizes: paragraphs 4-12; NNT: paragraph 13 |
|  | 17c | Report zero events if no harms were observed | Results, paragraph 16 |
| Ancillary analyses | 18 | Results of any other analyses performed, including subgroup analyses and adjusted analyses, distinguishing pre-specified from exploratory | Supplementary Data |
| Harms | 19 | All important harms or unintended effects in each group (for specific guidance see CONSORT for harms) | Results, paragraphs 13-14 |
| Discussion | | | |
| Limitations | 20 | Trial limitations, addressing sources of potential bias related to the approach to collecting or reporting data on  harms, imprecision, and, if relevant, multiplicity or selection of analyses | Discussion, paragraph 7 |
| Generalisability | 21 | Generalisability (external validity, applicability) of the trial findings | Discussion, paragraph 7 |
| Interpretation | 22 | Interpretation consistent with results, balancing benefits and harms, and considering other relevant evidence | Discussion, paragraph 1-5 |
| Other information | | |  |
| Registration | 23 | Registration number and name of trial registry | Abstract, Methods, paragraph 1 |
| Protocol | 24 | Where the full trial protocol and other relevant documents can be accessed, including additional data on harms | Methods, paragraph 1 |
| Funding | 25 | Sources of funding and other support (such as supply of drugs), role of funders | Methods, paragraph 16 |

Note: Adapted from Schulz (2010) to integrate items of CONSORT Harms 2022 (Junqueira 2022) [<https://creativecommons.org/licenses/by/2.0/>]. CONSORT items 1b, 2b, 6a, 11a, 12a, 13a, 14a, 16a, 17a, 17b, 18, 20 and 24 of were modified to incorporate elements relevant to the reporting of harms. Two new items were added (item 6c and 17a2). Please see the CONSORT Harms 2022 statement for additional details (Junqueira 2022).

We strongly recommend reading the CONSORT 2010 statement (Schulz 2010) in conjunction with the CONSORT Harms 2022 statement (Junqueira 2022) for important clarifications on all the items. If relevant, we also recommend reading CONSORT extensions for cluster randomised trials, non-inferiority and equivalence trials, non-pharmacological treatments, adaptive designs, pilot and feasibility studies, multi arm trials, cross-over and pragmatic trials. Additional extensions are forthcoming: for those and for up-to-date references relevant to this checklist, see [EQUATOR Network](https://www.equator-network.org/).

**References**

Junqueira DR, Zorzela L, Golder S, Loke Y, Gagnier JJ, Julious SA, Li T, Mayo-Wilson E, Pham B, Phillips R, Santaguida P, Scherer RW, Gøtzsche PC, Moher D, Ioannidis JPA and Vohra S on behalf of the CONSORT Harms Group. CONSORT Harms 2022 statement, explanation, and elaboration: updated guideline for the reporting of harms in randomised trials. *BMJ* 2023 **381:** e073725 DOI 10.1136/bmj-2022-073725

Schulz KF, Altman DG, Moher D, for the CONSORT Group. CONSORT 2010 Statement: updated guidelines for reporting parallel group randomised trials. *BMJ* 2010 **340:**c332 doi: 10.1136/bmj.c332.
